# Sirtuin E deacetylase is required for full virulence of Aspergillus fumigatus

**DOI:** 10.1038/s42003-024-06383-3

**Published:** 2024-06-08

**Authors:** Natália S. Wassano, Gabriela B. da Silva, Artur H. Reis, Jaqueline A. Gerhardt, Everton P. Antoniel, Daniel Akiyama, Caroline P. Rezende, Leandro X. Neves, Elton J. R. Vasconcelos, Fernanda L. de Figueiredo, Fausto Almeida, Patrícia A. de Castro, Camila F. Pinzan, Gustavo H. Goldman, Adriana F. Paes Leme, Taicia P. Fill, Nilmar S. Moretti, André Damasio

**Affiliations:** 1 Department of Biochemistry and Tissue Biology, Institute of Biology, University of Campinas (UNICAMP), Campinas, Brazil; 2 National Institute of Science and Technology in Human Pathogenic Fungi, Ribeirão Preto, Brazil; 3 Department of Microbiology, Immunology and Parasitology, Paulist School of Medicine, Federal University of São Paulo, São Paulo, Brazil; 4 Department of Organic Chemistry, Institute of Chemistry, University of Campinas (UNICAMP), Campinas, Brazil; 5 Department of Biochemistry and Immunology, Faculty of Medicine from Ribeirão Preto, University of São Paulo, São Paulo, Brazil; 6 Brazilian Bioscience National Laboratory (LNBio), Brazilian Center for Research in Energy and Materials (CNPEM), Campinas, Brazil; 7 Leeds Omics, Faculty of Biological Sciences, University of Leeds, Leeds, UK; 8 Faculdade de Ciências Farmacêuticas de Ribeirão Preto, Universidade de São Paulo, Ribeirão Preto, Brazil; 9 Department of Pathology and Microbiology, Faculty of Veterinary Medicine, University of Montreal, Saint-Hyacinthe, Canada; 10 The Research Group on Infectious Diseases in Production Animals (GREMIP), Faculty of Veterinary Medicine, University of Montreal, Saint-Hyacinthe, Canada

**Keywords:** Fungal biology, Pathogens

## Abstract

*Aspergillus fumigatus* represents a public health problem due to the high mortality rate in immunosuppressed patients and the emergence of antifungal-resistant isolates. Protein acetylation is a crucial post-translational modification that controls gene expression and biological processes. The strategic manipulation of enzymes involved in protein acetylation has emerged as a promising therapeutic approach for addressing fungal infections. Sirtuins, NAD^+^-dependent lysine deacetylases, regulate protein acetylation and gene expression in eukaryotes. However, their role in the human pathogenic fungus *A. fumigatus* remains unclear. This study constructs six single knockout strains of *A. fumigatus* and a strain lacking all predicted sirtuins (SIRTKO). The mutant strains are viable under laboratory conditions, indicating that sirtuins are not essential genes. Phenotypic assays suggest sirtuins’ involvement in cell wall integrity, secondary metabolite production, thermotolerance, and virulence. Deletion of *sirE* attenuates virulence in murine and *Galleria mellonella* infection models. The absence of SirE alters the acetylation status of proteins, including histones and non-histones, and triggers significant changes in the expression of genes associated with secondary metabolism, cell wall biosynthesis, and virulence factors. These findings encourage testing sirtuin inhibitors as potential therapeutic strategies to combat *A. fumigatus* infections or in combination therapy with available antifungals.

## Abstract (web summary)

Sirtuin’s function was studied in *A. fumigatus* by creating knockout strains. SirE encoding gene deletion reduces virulence, alters protein acetylation and gene expression. Sirtuin inhibitors have potential as therapy for *A. fumigatus* infections.

## Introduction

Humans are daily exposed to the opportunistic fungal pathogen *Aspergillus fumigatus*^1^. Although the immune system of healthy individuals is effective in eliminating this microorganism, immunosuppressed patients are at high risk of developing invasive pulmonary aspergillosis (IPA), a disease with a high mortality rate^2^. Moreover, the emergence of COVID-19-associated IPA and antifungal-resistant isolates has raised serious medical concerns^3,4^. IPA pathogenesis depends on multiple host factors such as the immune system status^1,5–7^. Moreover, some characteristics of *A. fumigatus* are essential factors for full virulence, such as (i) conidial melanin and hydrophobicity that protect against reactive oxygen species (ROS) and damage by immune cells^8,9^, (ii) the ability to survive at high temperature^10^, and (iii) production of secondary metabolites (SMs), such as siderophores and gliotoxin^11,12^.

Previous studies indicate that chromatin conformation plays a crucial role in metabolism, development, and differentiation, as well as in fungal virulence and host–pathogen interactions^13–15^. Moreover, post-translational modifications (PTMs) in histones and non-histone proteins, such as acetylation, methylation, and phosphorylation, have fundamental roles in regulating several cellular processes ranging from cancer to infectious diseases^16,17^. In particular, histone acetylation, which usually occurs at lysine residues at the N-terminus of histone proteins, is generally associated with transcriptional activation promoting euchromatin conformation. In contrast, histone deacetylation is usually related to transcriptional repression by heterochromatin conformation^18,19^. The acetylation of lysine residues is regulated by lysine acetyltransferases (KATs) and non-enzymatic reactions^18^. The opposite reaction is controlled by lysine deacetylases, or KDACs, which remove an acetyl group from lysine residues^20,21^. The classical KDACs are Zn^2+^-dependent, whereas sirtuins are NAD^+^-dependent deacetylases. Together with KATs, they maintain a balanced acetylation status of proteins.

Recently, acetylation of histone and non-histone proteins has been investigated for its role in regulating gene expression, developmental processes, metabolism, stress resistance, pathogenesis, and virulence in some filamentous fungi^22–39^. In the human pathogen *A. fumigatus*, SasC and Rtt109 proteins among the KATs studied have been found to play roles in growth, asexual development, spore germination, stress response, and virulence^40,41^. On the other hand, classical KDACs such as HdaA and RpdA have been reported to be essential for producing secondary metabolites, and cell viability and virulence, respectively^34,42^. Although sirtuins regulate fungal growth, sporulation, stress responses, production of secondary metabolites and virulence in some *Aspergillus* species^32,36–38,43^, their biological role in the human pathogen *A. fumigatus* remains unexplored.

This study aims to fill this knowledge gap by examining the role of sirtuins in *A. fumigatus* and their impact on key biological processes and virulence. In silico analysis indicated that *A. fumigatus* has six genes encoding putative sirtuins and their deacetylase activity was confirmed by in vitro assays using recombinant proteins. By combining gene knockout and extensive phenotyping of single mutant strains (Δ*sirA*, Δ*sirB*, Δ*sirC*, Δ*sirD*, Δ*sirE*, and Δ*hstA*), together with transcriptomic profiling, we demonstrate that despite sirtuins are not essential for *A. fumigatus* survival, they are involved in several biological processes. Our findings indicate their involvement in virulence, thermotolerance, SM production, and cell wall biosynthesis. Additionally, acetylome data obtained from a single mutant strain (∆*sirE*) and the null sirtuins strain (SIRTKO) provided valuable insights into the direct or indirect regulation of acetylation balance by sirtuins. Significant changes in the acetylation status of numerous proteins were observed, including histones and proteins encoded by genetic determinants of virulence. These findings demonstrate the importance of sirtuins in *A. fumigatus* biology and pathogenesis. Understanding the role of sirtuins in this human pathogenic fungus may pave the way for effectively developing novel therapeutic strategies to combat *A. fumigatus* infections, particularly in immunocompromised patients.

## Results

### *A. fumigatus* harbors six sirtuin genes

**Fig. 1:**
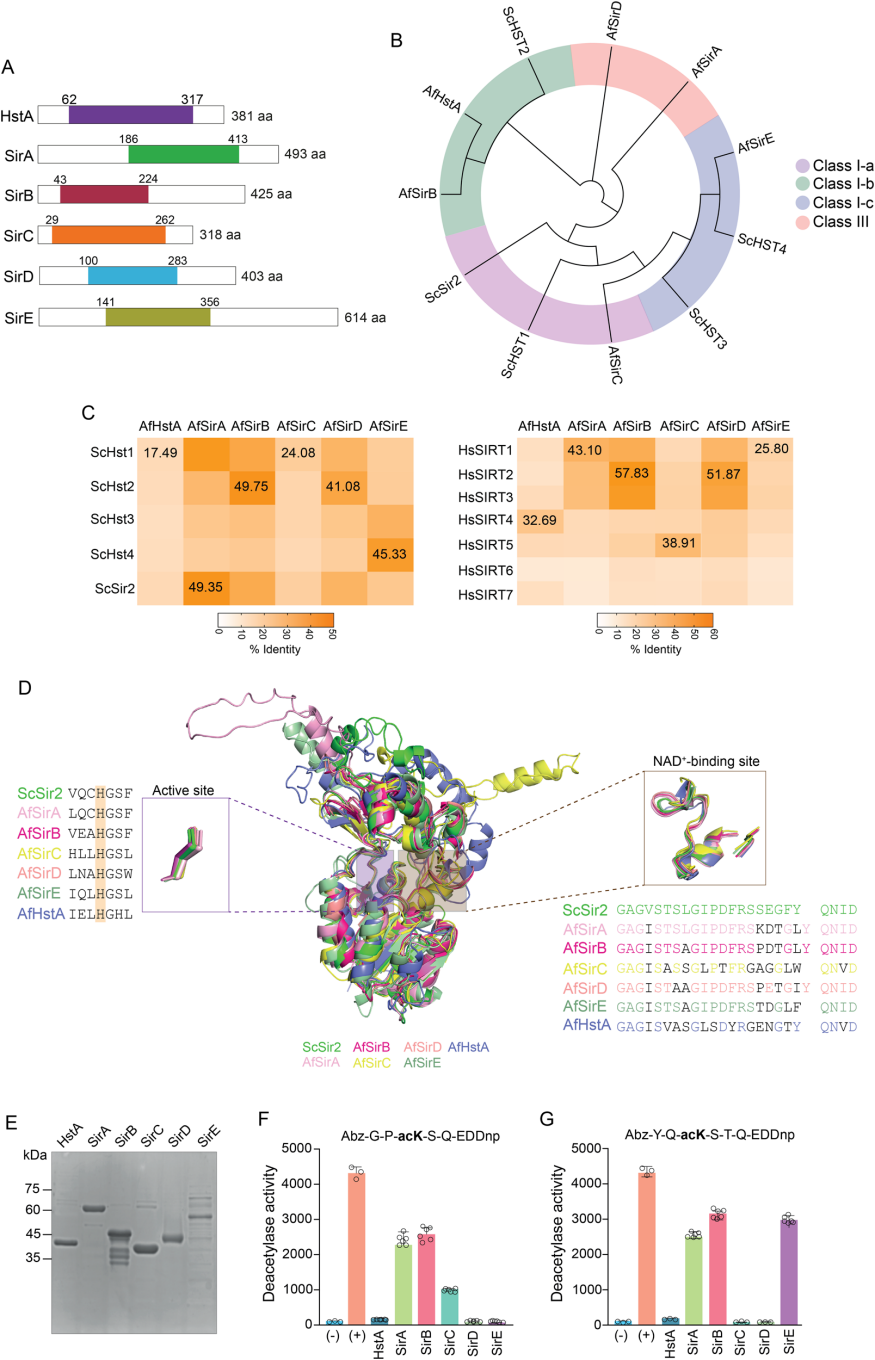
*A. fumigatus* sirtuins are active deacetylases. **A** In silico analysis showed that *A. fumigatus* harbors six schematically represented sirtuins. The sirtuin catalytic domains are colored and flanked by distinct N- and C-terminal extensions. The numbers below each domain indicate the amino acid position for orientation. **B** Phylogenetic tree of *A. fumigatus* (Af) and *S. cerevisiae* (Sc) sirtuins. The sirtuins are divided into Class Ia, Ib, II, and III for both species. **C** Identity (%) of sirtuin domains among *A. fumigatus*, *Saccharomyces cerevisiae* and human. The alignment of full sequences is depicted in Supplementary Fig. 1. **D** Tridimensional structural analyses of predicted *A. fumigatus* sirtuin domains compared to *S. cerevisiae* homologs indicating the catalytic site with conserved histidine and NAD+ binding region. **E** SDS–PAGE gel of purified *A. fumigatus* recombinant sirtuins used in the in vitro deacetylation assays. Expected sizes for each protein: AfHstA (41.5 kDa); AfSirA (54.6 kDA); AfSirB (46.9 kDa); AfSirC (34.6 kDa); AfSirD (45.1 kDa); AfSirE (67.1 kDa). **F** and **G** In vitro deacetylase activity of *A. fumigatus* recombinant sirtuins using a-tubulin K40ac (**F**) and histone H3K56ac (**G**) peptides as substrates. HstA and SirD were the only enzymes that did not present deacetylase activity. The positive control used in the assays was the *Trypanosoma cruzi* Sir2rp1.

Six putative genes encoding sirtuins were identified in *A. fumigatus* genome (FungiDB) using the SirA sequence from *A. nidulans* (AN10449) as a query. These six proteins have the characteristic sirtuin domain and are grouped into four classes (Class Ia, Ib, Ic, and III), as well as *S. cerevisiae* orthologs (Fig. 1A, B). The analyses of amino acid identity of *A. fumigatus* sirtuins (full length and sirtuin domain) showed a degree of conservation ranging from 17% to 49% and 25% to 57% of identity considering the sirtuin domain relative to *S. cerevisiae* and human orthologs, respectively (Fig. 1C and Supplementary Fig. 1A). Moreover, amino acid sequence analyses demonstrated the presence of *A. fumigatus* sirtuin orthologues in several *Aspergillus* species (human pathogenic or not) with a high degree of conservation (Supplementary Fig. 1B, C).

To gain insight into the characterization of *A. fumigatus* sirtuins, 3D predicted structural models for all enzymes were generated using the AlphaFold tool. All predicted models presented high confidence (Supplementary Fig. 2) and were used for comparative structural analyses with *S. cerevisiae* Sir2. The sirtuin domain of all *A. fumigatus* proteins has a high structural conservation degree compared to *S. cerevisiae* enzyme, including the key histidine involved in enzymatic activity and the residues of NAD^+^-binding site (Fig. 1D).

To validate the deacetylase activity of *A. fumigatus* sirtuins, the six sirtuin-encoding genes were expressed in *E. coli* and used to test in vitro deacetylase activity (Fig. 1E). The deacetylase activity of *A. fumigatus* sirtuins was measured using acetylated peptides as substrates, histone H3K56ac from *A. nidulans* (YQacKSTQ)^36^ and ɑ-tubulin K40ac (GPacKSQ). Recombinant sirtuins showed in vitro deacetylase activity, except AfHstA and AfSirD (Fig. 1F, G). Interestingly, while AfSirA and AfSirB were active on both peptides, AfSirC was active only in the ɑ-tubulin K40ac (Fig. 1F), and AfSirE showed specificity for the H3K56ac peptide (Fig. 1G), demonstrating a substrate-dependent activity of *A. fumigatus* sirtuins.

### Sirtuin-encoding genes are not essential to *A. fumigatus* but are involved in cell wall integrity, thermotolerance, and antifungal sensibility

**Fig. 2:**
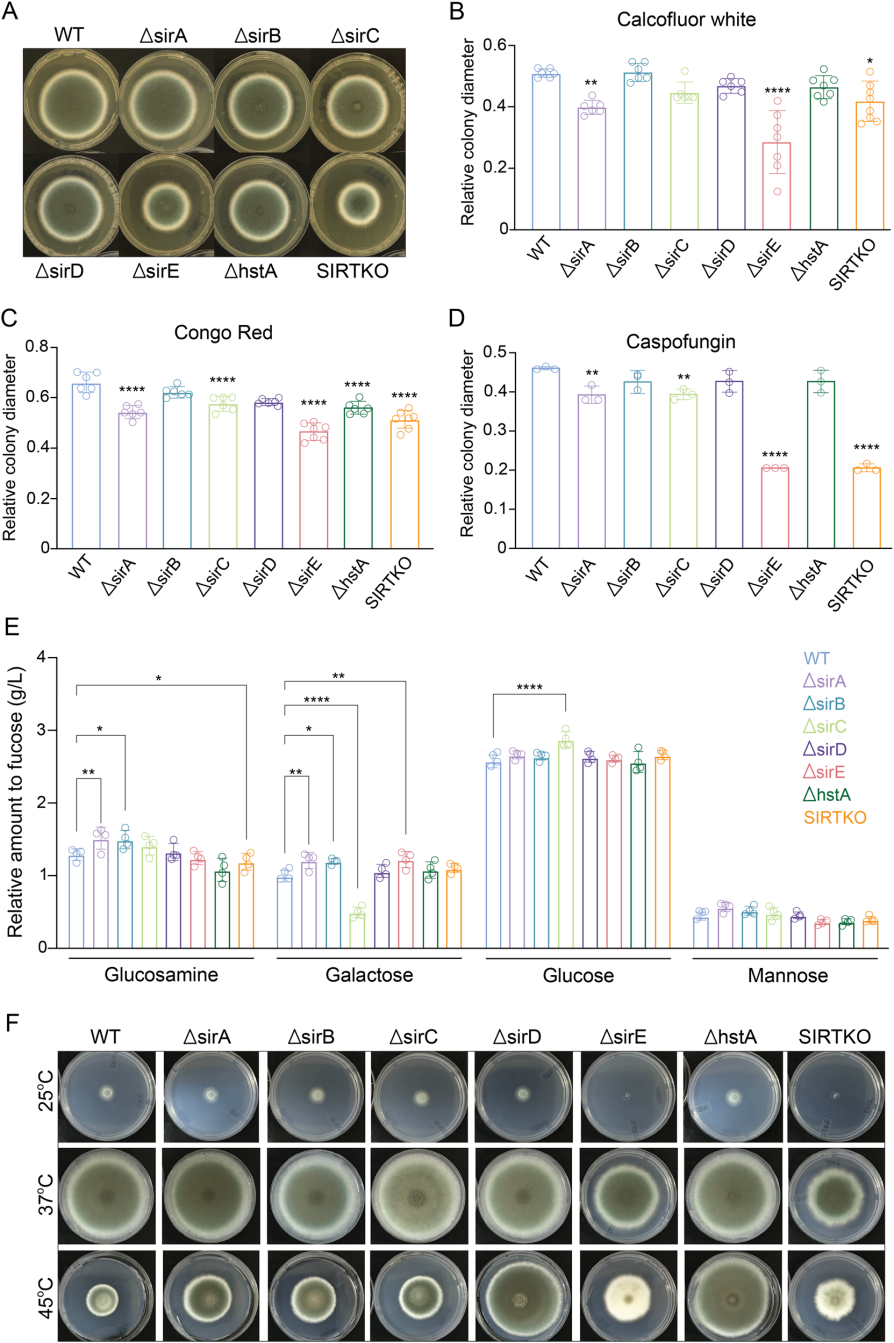
Phenotypic characterization of *A. fumigatus* sirtuin mutants. **A** *A. fumigatu*s mutants inoculated on YAG medium for 120 h at 37 °C. **B** Relative quantification of mutant strains growth based on colony diameter in the presence of calcofluor white (30 µg/mL, *n* = 6), **C** congo red (50 µg/mL, *n* = 6), and **D** caspofungin (1 µg/mL, *n* = 3). **E** Relative quantification based on feature area of cell wall monosaccharides (glucose, mannose, glucosamine, and galactose) from WT and sirtuin mutant mycelia after acid (H_2_SO_4_) treatment (*n* = 3). 1 mg/mL of fucose was used as an internal control for normalization. **F** *A. fumigatus* sirtuin mutants grown on glucose minimal media (GMM) for 120 h at different temperatures. Significant differences were observed by using two-way ANOVA. *p < 0.05, **p < 0.002, ***p < 0.001 and ****p < 0.0001 indicate significant differences from comparisons to the WT strain.

Six *A. fumigatus* single knockout strains (Δ*sirA*, Δ*sirB*, Δ*sirC*, Δ*sirD*, Δ*sirE*, and Δ*hstA*) in addition to a strain carrying the deletion of the six sirtuins (SIRTKO) were constructed using the CRISPR-Cas9 system available for *Aspergilli*^44,45^. The deletions were confirmed by PCR and Southern blot analyses (Supplementary Figs. 3 and 4). All the single mutants were viable under lab conditions; however, ∆*sir*E and SIRTKO displayed significant growth defects in yeast agar glucose (YAG) medium (Fig. 2A). The influence of sirtuins on cell wall integrity was evaluated by exposing the mutant strains to cell wall stressors. ∆*sirA*, ∆*sirB*, ∆*sirE*, and SIRTKO strains showed sensitivity to cell wall stressors, such as Congo Red (CR), Calcofluor White (CFW), and caspofungin, compared to the WT (A1160^-^) (Fig. 2B–D). Moreover, analyzing the monosaccharides in the cell wall revealed significant differences in the contents of glucosamine in ∆*sirA*, ∆*sirB*, and ∆*hstA* strains and galactose in ∆*sirA*, ∆*sirB*, ∆*sir*C, and ∆*sirE* mutants compared to the WT strain, but no differences in mannose content (Fig. 2E). In addition, all sirtuin mutants resisted high temperatures (Fig. 2F). The ∆*sirE* and SIRTKO strains demonstrated greater sensitivity to caspofungin and voriconazole incorporated into a solid medium. In addition, complete inhibition of macroscopic growth was observed at 2 μg/mL of caspofungin, rather than partial inhibition defined as the minimal effective concentration (MEC) for caspofungin (Supplementary Fig. 5A and B).

### *A. fumigatus* sirtuins are necessary for full virulence

**Fig. 3:**
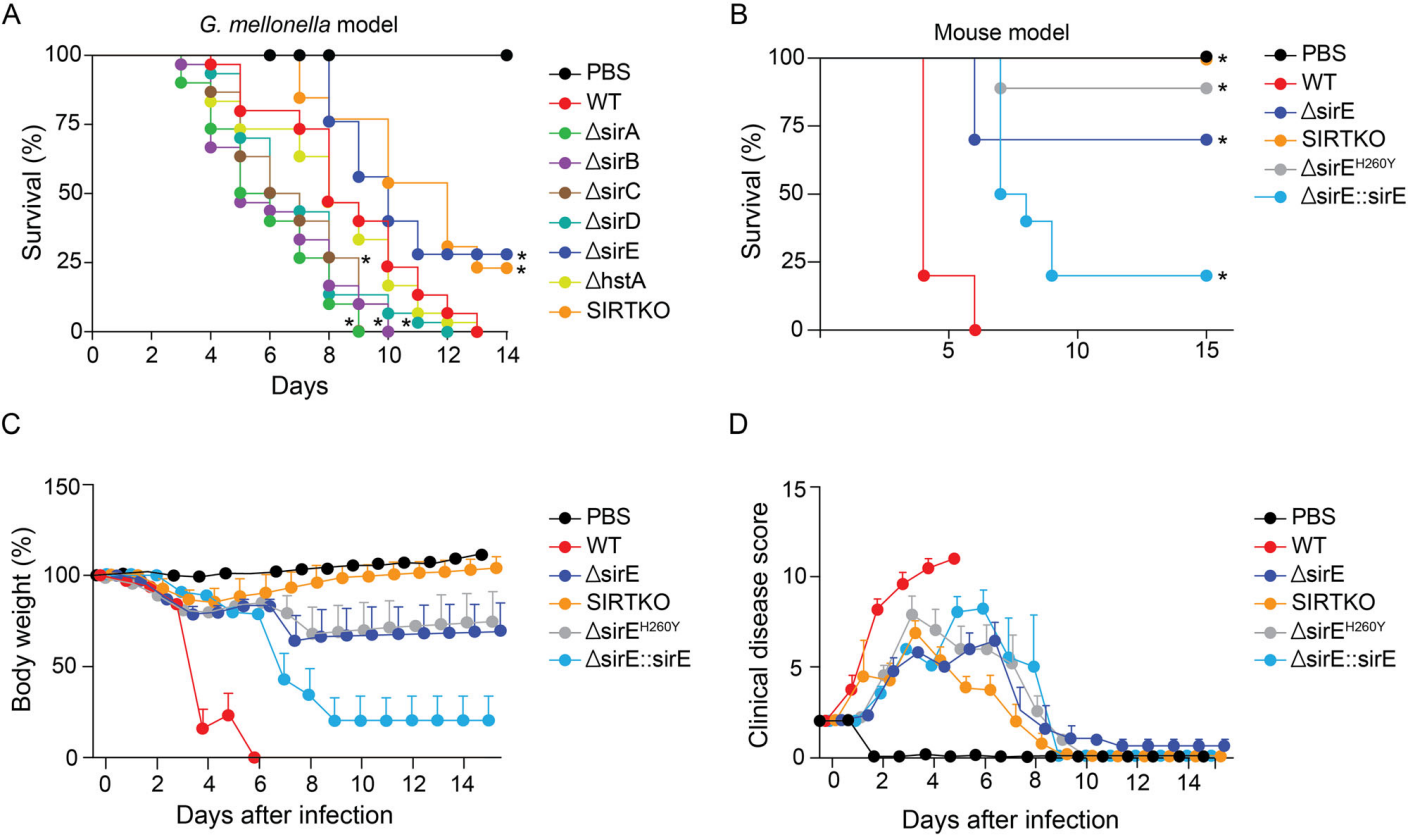
Virulence assay. **A** Survival curve of *A. fumigatus* sirtuin mutant strains using *Galleria mellonella* model. The graph represents the average of three independent replicates using 10 larvae for each experiment. Significant differences were observed by using a two-way ANOVA *p < 0.05. **B** Survival curve of WT, ∆*sirE*, sirE^H260Y^_,_ and ∆sirE_:_:sirE complemented strains in female BALB/c mice (*n* = 10). The statistical significance of comparative survival values was calculated by the Prism statistical analysis package by using Log-rank (Mantel–Cox) Test and Gehan–Brestow–Wilcoxon tests. **C** Body weight variation of infected animals with the different *A. fumigatus* strains. **D** Clinical Disease Score of animals during the course of infection. For all variables, normal distribution and homogeneous variance were tested. The differences observed were considered significant when *p* < 0.05.

To access the virulence profile of sirtuin mutant strains, we reinserted the native *pyrG* gene in each mutant strain due to the avirulent profile of uracil/uridine auxotroph strains^46^. *G. mellonella* larvae were infected with the conidia of each mutant strain, and their survival was evaluated. Interestingly, the ∆*sirA*, ∆*sirB*, ∆*sirC* and ∆*hstA* strains were hypervirulent and the ∆*sirE* and SIRTKO strains were less virulent when compared to the WT strain (Fig. 3A). These results indicate that sirtuins are necessary for full *A. fumigatus* virulence in a non-vertebrate model. Due to the attenuated virulence of *∆sirE* strain, an additional assay was carried out using a murine model (Fig. 3B). To do that, we constructed a *sirE* complemented strain (∆*sirE*::*sirE*) and a strain carrying a mutated version of SirE, substituting a histidine (H) at position 260 with tyrosine (Y) (*sirE*^H260Y^) by site-directed mutagenesis (Fig. 1B). Virulence of ∆*sirE*, *sirE*^H260Y^ was attenuated in the neutropenic mouse model, while the SIRTKO strain was avirulent. The WT virulence was partially recovered in the complemented strain ∆*sirE*::*sirE*, indicating that loss of SirE activity resulted in a severe alteration in protein acetylation. The reduced SirE^H260Y^ activity was confirmed by testing the in vitro deacetylation compared to the native protein (Supplementary Fig. 6). Compared to the WT strain, the mutant strains SIRTKO, ∆*sirE*, and sirE^H260Y^ showed less severe body weight loss (Fig. 3C) and clinical signs (Fig. 3D) in line with the survival curve.

### Sirtuins regulate secondary metabolite production

**Fig. 4:**
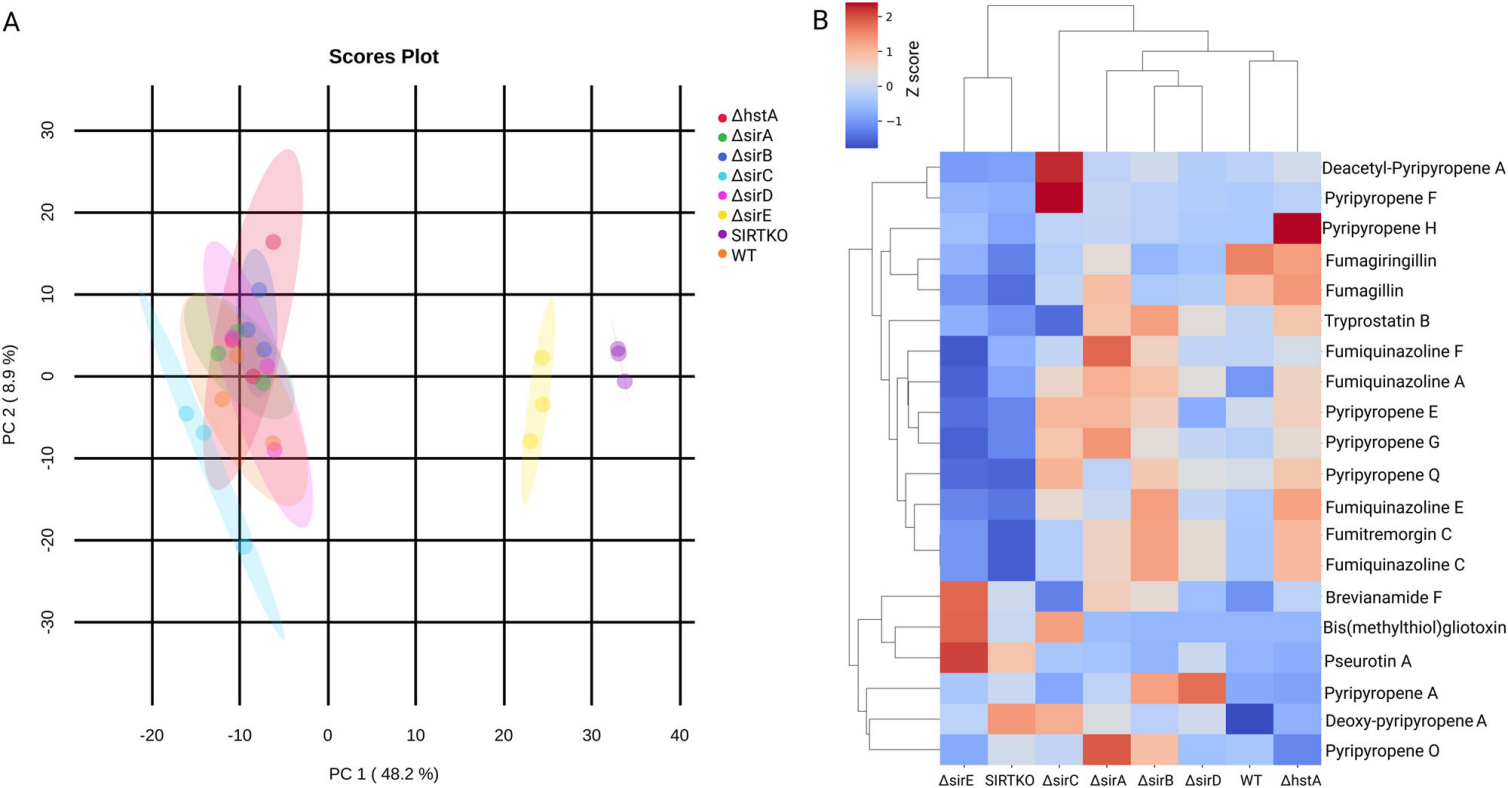
Secondary metabolite profile of *A. fumigatus* sirtuin mutant strains. **A** Principal component analysis (PCA) of the metabolic extracts of *A. fumigatus* sirtuin mutant strains. PCA model after removal of QC, analytical blanks, and GMM media samples. **B** Relative quantification of annotated secondary metabolites based on feature area and normalized by *z*-score. Data was acquired from an average of three biological replicates.

The metabolome of sirtuin knockout strains was analyzed to understand the impact of sirtuins on the regulation of SMs in *A. fumigatus*. Principal component analysis (PCA) was applied to examine data acquisition reproducibility, batch carry-over effects, and chemical-based grouping tendencies (Supplementary Fig. 7A). After ensuring the clustering of QC samples near the origin of the coordinate system, which indicates good analytical method reproducibility, a PCA model was constructed with the samples of only the fungal strains (Fig. 4A). The 2D score plot (Supplementary Fig. 7B) illustrates the clustering of WT, ∆*hstA* and ∆*sirA-D* groups, indicating that a small metabolic variation resulted from the knockout of these genes. On the other hand, the metabolite profile of SIRTKO and ∆*sirE* was distinct, as observed across principal component 1. To identify SMs altered in SIRTKO and ∆*sirE*, structural annotation of chemical features was performed by either querying acquired MS/MS spectra against the Global Natural Products Social Molecular Networking (GNPS) public database or propagating annotations across molecular networks based on accurate mass and fragmentation similarity of well-established *A. fumigatus* SMs. Relative quantification based on the feature area of annotated SMs is represented on a heatmap (Fig. 4B and Supplementary Fig. 8). The data indicated that Pseurotin A, Brevinamide F, Pyripyroropene A, Deacetyl-Pyriopyropene and Bis(methylthio)gliotoxin were more abundant in both SIRTKO and Δ*sirE* strains, while Fumiquinazoline E and C, Trypostin B, Pyripiropene E-H, and Fumagillin were found in low abundance compared to the WT strain (Fig. 4B). These data demonstrate that sirtuins affect the production of SMs in *A. fumigatus*.

### Deletion of sirtuin affects the abundance of protein acetylation

Quantitative acetylome of the attenuated virulent strains (∆*sirE* and SIRTKO) was performed by enrichment of acetylated peptides using the PTMScan® Acetyl-Lysine Motif [Ac-K] Kit (Cell Signaling Technology). The intracellular proteins of WT and mutant strains were extracted, then digested with trypsin, and either the total proteome and acetyl-lysine (Kac)-enriched fraction were analyzed by nLC-MS/MS.

**Fig. 5:**
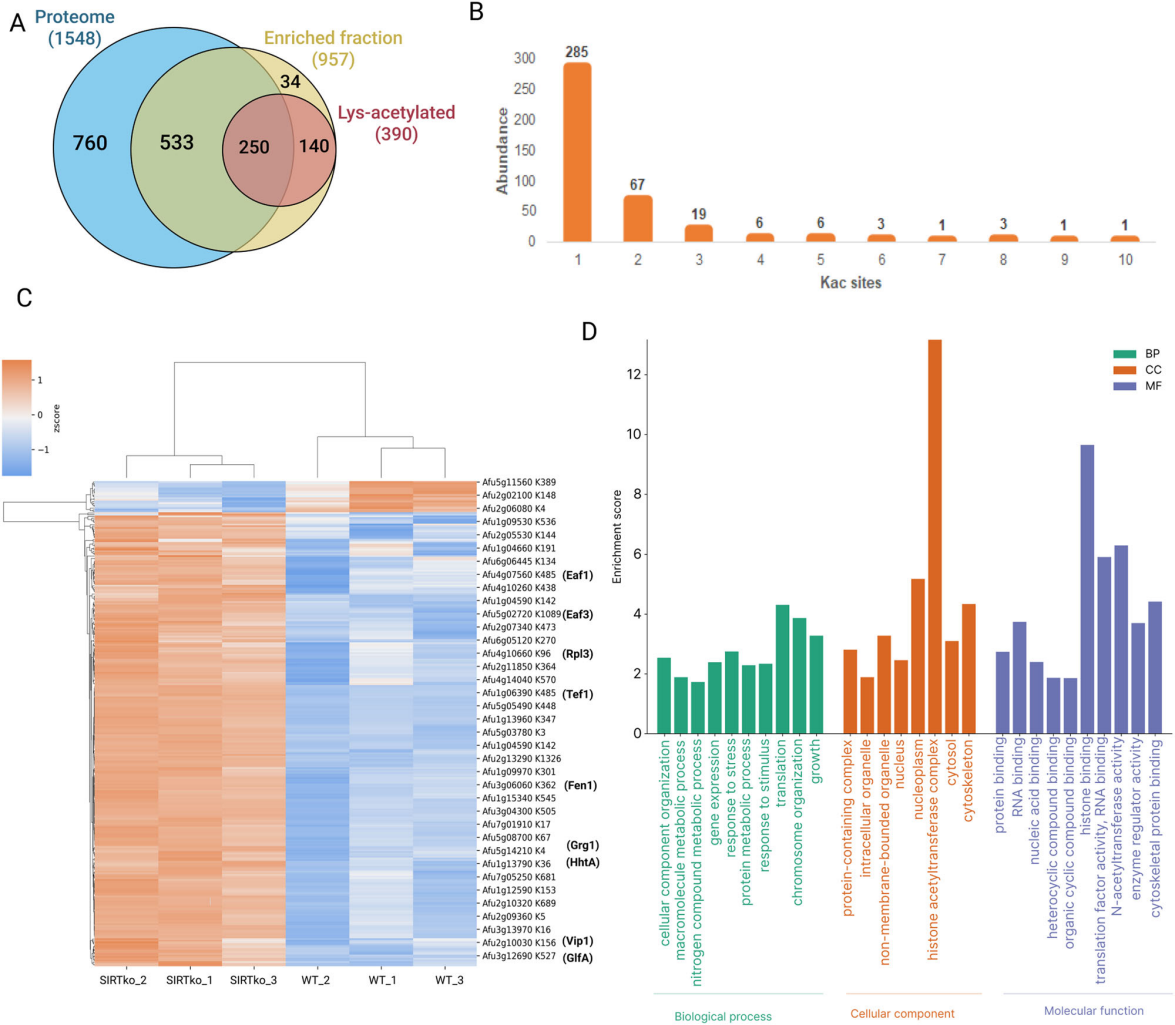
Acetylome analysis of *A. fumigatus* SIRTKO strain. **A** Venn diagram of proteins found in the total proteome, enriched fraction, and lysine-acetylated proteins of WT and SIRTKO strains. **B** Distribution of the number of acetylated sites (Kac) in the proteome. **C** Heat map of peptides containing Kac-sites showing changes in abundance when WT and SIRTKO are compared (FDR adjusted *t*-test < 0.05). Kac-peptide intensities were *z*-score normalized, and Euclidean distance hierarchical clustering was applied to rows and columns. **D** GO enrichment of the 216 proteins with differentially abundant Kac sites was performed using FungiDB and visualized on the SRPlot website.

A total of 1548 proteins, or 15.7% of the *A. fumigatus* proteome, were identified in the SIRTKO and WT strains, while 957 proteins were found in the Kac-enriched fraction, with 390 of those proteins containing 598 Kac sites (Fig. 5A). Our findings showed that 72.7% of the acetylated proteins contained one Kac site, while 27.3% were acetylated at two or more lysine residues (Fig. 5B). A total of 159 Kac sites were distributed among 118 acetylated proteins in the WT strain, while 318 acetylated proteins displayed 439 Kac sites in the SIRTKO strain. Moreover, 295 Kac sites were found differentially acetylated in 216 proteins (False Discovery Rate (FDR) < 0.5; Fig. 5C and Supplementary Data 1). Among them, we found different abundances in histone acetylation. Histone H3 was abundantly acetylated at positions H3K56, H3K9, H3K36, H3K27, and H3K14, while lower acetylation in histone H2B was detected at position H2BK14, H2BK7, H2BK19, H2BK24 in the SIRTKO strain. The set of differentially acetylated proteins was used as input for Gene Ontology (GO) enrichment analysis (Fig. 5D), resulting in some overrepresented biological processes (BP) such as regulation of cellular component organization (GO:0016043), gene expression (GO:0010467), response to stress (GO:0033554), macromolecule metabolic process (GO:0019222), growth (GO:0040007) and others. The enriched cellular components (CC) range from the nucleus to the cell wall, whereas for molecular function (MF) there were enriched terms such as translation factor activity, RNA and histone binding, and N-acetyltransferase activity (Fig. 5D and Supplementary Data 1). Seven proteins encoded by genetic determinants of virulence were abundantly acetylated in the SIRTKO acetylome. These proteins are involved in metabolism, cell wall integrity, and signaling, such as the Bzip developmental regulator (Afu2g14680/flbB), putative mitogen-activated protein kinase kinase kinase—MAPKKK (Afu3g1108/bck1), UDP-galactopyranose mutase (Afu3g12690/glfA), cross-pathway control WD-repeat protein (Afu4g13170/ cpcB), GTP-Binding nuclear protein (Afu6g13300/Ran), and putative septin (Afu7g05370/aspB).

In addition to the protein acetylation profile, 148 proteins were differentially expressed in the SIRTKO strain relative to the WT strain (Supplementary Data 2). These proteins are enriched for the nucleotide metabolic process (GO:0009117), response to stress (GO:0006950), small molecule metabolic process (GO:0044281), and other biological processes (Supplementary Data 2).

**Fig. 6:**
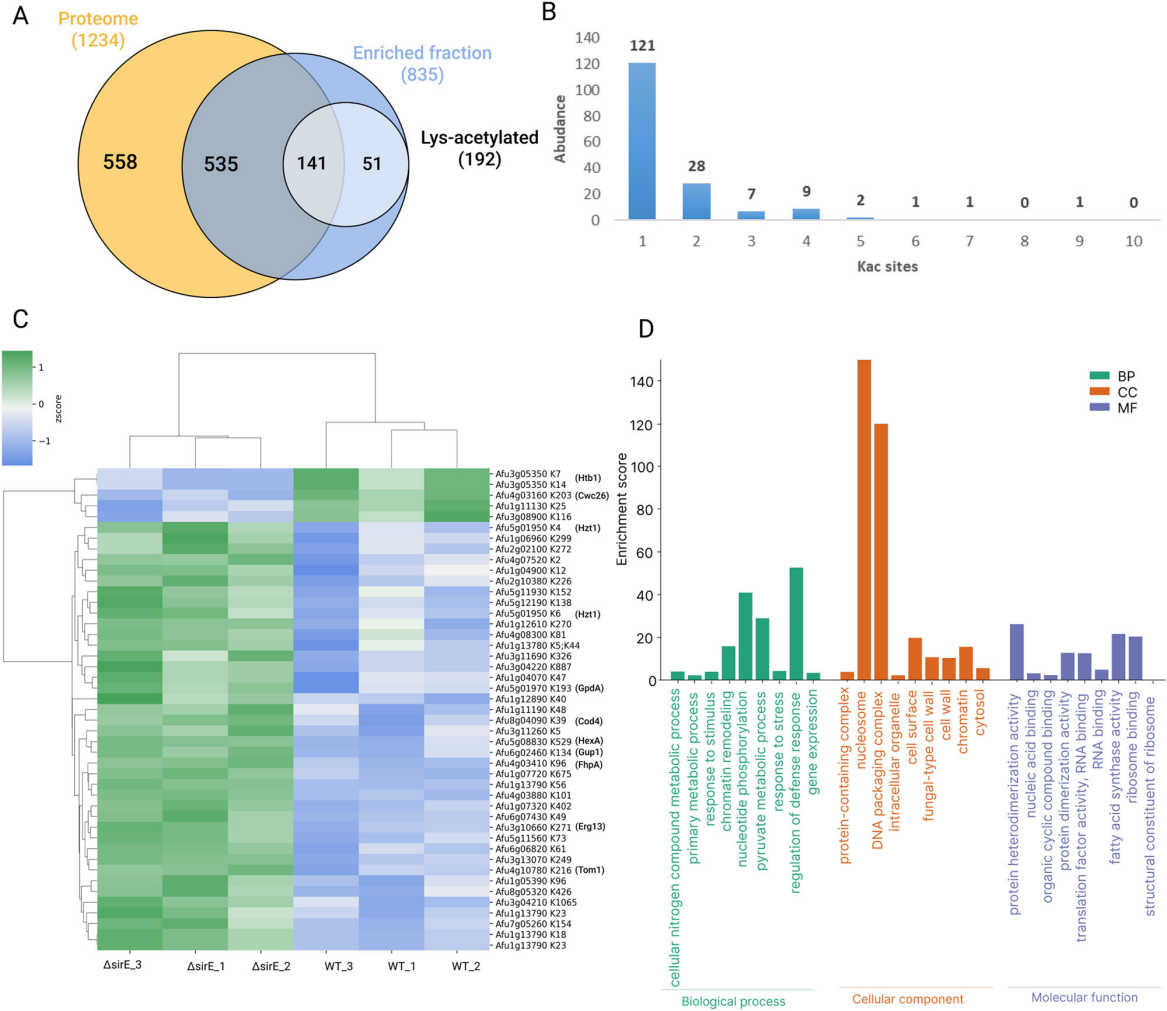
Acetylome analysis of *A. fumigatus* ∆*sirE* strain. **A** Venn diagram of proteins found in the total proteome, enriched fraction, and lysine-acetylated proteins of WT and ∆*sirE* strains. **B** Distribution of the number of acetylated sites (Kac) in the proteome. **C** Heat map of peptides containing Kac-sites showing changes in abundance when WT and ∆*sirE* are compared (FDR adjusted *t*-test < 0.05). Kac-peptide intensities were *z*-score normalized, and Euclidean distance hierarchical clustering was applied to rows and columns. **D** GO enrichment analysis of the 40 proteins with differentially abundant Kac sites was performed using FungiDB and visualized on the SRPlot website.

Around 1390 proteins were detected in the ∆*sirE* and WT proteomes, and 835 proteins were identified in the enriched fraction, in which 262 Kac sites were distributed among 192 proteins (Fig. 6A). Approximately 71% of these acetylated proteins have only one acetylation site (Fig. 6B). Moreover, 46 Kac sites were found differentially acetylated in 40 proteins of the ∆*sirE* strain (Fig. 6C). GO terms enrichment analysis indicated that these proteins are involved with the regulation of defense response (GO:0031347), chromatin remodeling (GO:0006338), pyruvate metabolism (GO:0006090), as well as the GO terms cytosol (GO:0005829), DNA packing complex (GO:0044815), protein-containing complex (GO:0032991), cell wall (GO:0005618), binding (GO:0005488), and fatty acid synthase activity (GO:0004312) were also enriched (Fig. 6D and Supplementary Data 1). Histones H3, H2A, and H4 were abundantly acetylated (H3K56; H3K18; H3K23; H2AK6; H2AK4; H4K5) in the Δ*sirE* acetylome compared to the WT strain, while a lower acetylation abundance was found for histone H2B (H2BK7; H2BK14). In addition, 61 proteins were differentially expressed in the ∆*sirE* total proteome, in which 42 hits were upregulated and 19 downregulated (Supplementary Data 2). These differentially expressed proteins are enriched for pyrimidine deoxyribose metabolic and biosynthetic process, stress response, COPII-coated vesicle cargo loading, fatty acid metabolic process, and homocysteine biosynthesis (Supplementary Data 2).

### Sirtuins regulate the transcription of genetic determinants of virulence

**Fig. 7:**
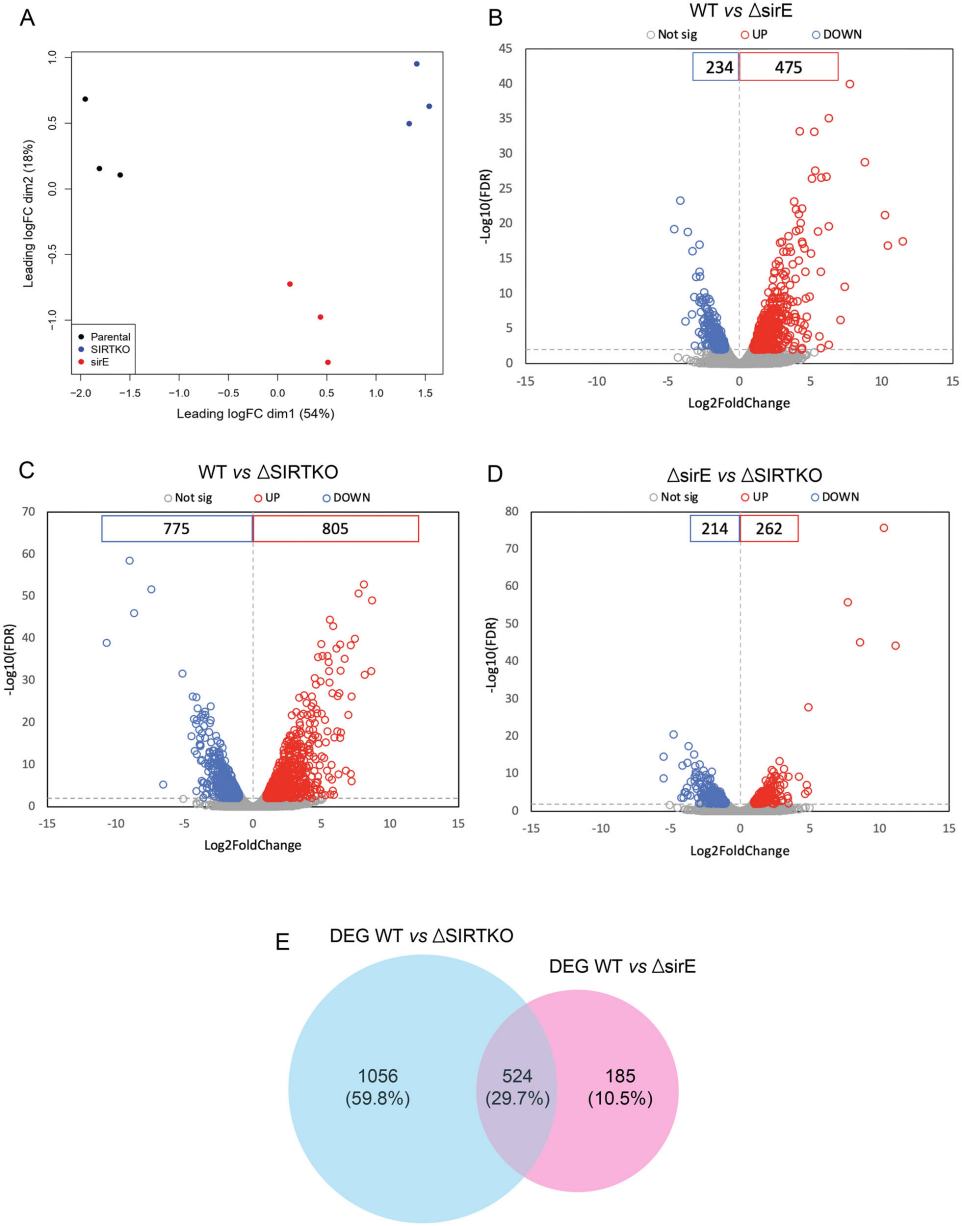
Transcriptome analysis of *A. fumigatus* WT (A1160-), Δ*sirE* and SIRTKO strains. **A** Multi-dimensional scaling (MDS) plot of RNA-seq datasets. **B–D** Volcano plot of differentially expressed genes (|log2(FC)| > 1 and FDR < 1e^−5^). The upregulated genes are labeled in red, while the downregulated are labeled in blue. **E** Venn diagram comparing the differentially expressed genes (DEG) among WT vs. SIRTKO and WT vs. Δ*sirE*.

To understand whether sirtuins regulate gene expression, a comparative transcriptome analysis of WT, Δ*sirE* and SIRTKO strains cultured on GMM medium for 36 h was performed. The multi-dimensional scaling (MDS) plot of RNA-seq datasets and volcano plot of differentially expressed genes (DEGs) show a variation among samples (Fig. 7A). We identified 709 DEGs on the Δ*sirE* strain (FDR ≤ 1e^−2^, mean Log2(fold change) ≥ 1 and ≤ −1), of which 475 were upregulated and 234 downregulated compared to the WT strain (Fig. 7B). On the SIRTKO strain, 1580 DEGs were identified compared to WT, with 805 up- and 775 downregulated (Fig. 7C). 476 DEGs were identified comparing SIRTKO and Δ*sirE*, among them 262 were upregulated and 214 downregulated (Fig. 7D). Deletion of *sirE* resulted in 29.7% of DEGs overlapping with SIRTKO, indicating strong regulation of gene expression among other sirtuins (Fig. 7E).

**Fig. 8:**
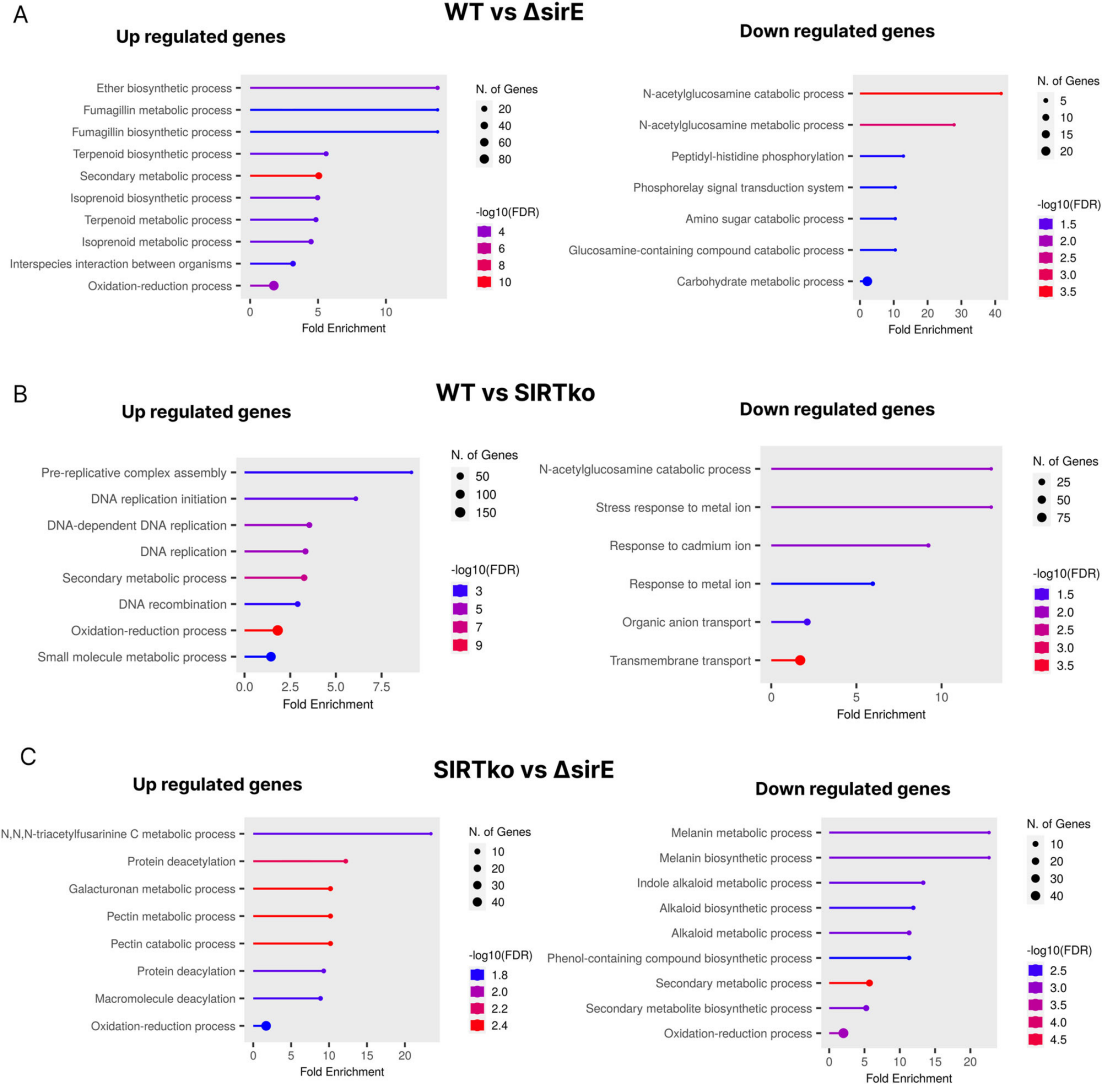
Transcriptome analysis of *A. fumigatus* WT (A1160-), Δ*sirE* and SIRTKO strains. Gene ontology enrichment for the biological function of upregulated and downregulated genes using the ShinyGO web platform for data visualization (http:/bioinformatics.sdstate.edu/go/). **A** WT versus Δ*sirE*, **B** WT versus SIRTKO and **C** SIRTKO versus Δ*sirE*.

There are 34 genes potentially involved in *A. fumigatus* virulence upregulated in the ∆*sirE* strain (Supplementary Data 3). These genes are involved in the biosynthesis of SMs, such as gliotoxin, fumagillin, brevianamide F, verruculogen, and fumitremorgin. Several putative cytochrome p450 monooxygenase enzymes (CYP450), which contribute to the production and diversity of various SMs^47^ were also upregulated in the Δ*sirE* strain. Among downregulated genes in this strain, six are determinants of virulence, such as sensor histidine kinase/response regulator (Fos-1/TcsA; Afu6g10240), also found downregulated in response to itraconazole^48^, conidial hydrophobin (rodA; Afu5g09580), putative transporter (hasB; Afu3g12900), and the C6 transcription factor hasA (Afu3g12890) which controls the production of hexadehydroastechrome^49^. GO enrichment analysis indicated that among upregulated genes, the most enriched biological process was associated with secondary metabolic processes (GO:0019748) (Fig. 8A and Supplementary Data 3 and 4). The downregulated genes were associated with transmembrane transport (GO:0072348), N-acetylglucosamine metabolic process (GO:0006044), glucosamine catabolic process (GO:0006043), and amino sugar catabolic process (GO:0046348) (Fig. 8A and Supplementary Data 3 and 4).

Among the DEGs in the SIRTKO strain, 33 and 18 genetic determinants of virulence were up and downregulated, respectively. There are several genes involved in SMs biosynthesis, such as heptaketide hydrolyase (*ayg1*/Afu2g17550), putative fatty acid oxygenase (Afu3g12120), conidial pigment polyketide synthase (*alb1*/Afu2g17600), as well as putative acetyltransferase with a predicted role in iron metabolism (Afu3g03650) upregulated in the SIRTKO strain. Among the 18 downregulated genes, there are transcription factors involved in the regulation of morphogenesis, gliotoxin production, and virulence (Afu6g02690/*mtfA*), putative chitin synthase (Afu8g05630/*chsF*), putative regulator of adherence, host cell interactions and virulence (Afu2g13260/*medA*), and putative mitogen-activated protein kinase (Afu5g09100/*mpkC*).

GO enrichment analysis of upregulated genes showed DNA replication (GO:0006260), and secondary metabolic process (GO:0019748) as enriched biological processes (Fig. 8B and Supplementary Data 3 and 4), while the downregulated genes are involved in transmembrane transport (GO:0055085), N-acetylglucosamine catabolic process (GO:0006046), and regulation of stress-activated MAPK cascade (GO:0032872) (Fig. 8B and Supplementary Data 3 and 4).

To get further insights into the specific roles of SirE and the other sirtuins, some analysis was conducted by mixing the data from Δ*sirE* and SIRTKO mutants. The enriched GO terms of DEG among SIRTKO versus Δ*sirE* showed upregulation of protein deacetylation due to the absence of additional sirtuins in the SIRTKO strain. Interestingly, galacturonan and pectin metabolic processes (GO:0010393; GO:0045488) were enriched among upregulated genes in Δ*sirE*, suggesting that the other sirtuins have an important role in the production of enzymes involved in polysaccharide degradation. The downregulated genes are enriched for melanin and secondary metabolic processes (GO:0006582, GO:0019748), indicating that the deletion of all six sirtuins increases the expression of genes related to secondary metabolite biosynthesis (Fig. 8C).

In summary, combining the transcriptome and acetylome analysis with the in vitro deacetylase activity, our findings suggest that sirtuin E loss-of-function leads to an increase in histone H3 acetylation, which could potentially trigger the upregulation of a large set of genes. It is still unclear whether the decrease in virulence resulted from a growth defect, significant alterations in gene expression due to SirE inactivity, a severe alteration in protein acetylation, including proteins coded by virulence-related genes, or a combination of these factors.

## Discussion

Protein acetylation has been correlated to fungal growth, metabolism, pathogenesis, and SM production^50–52^. The balance of acetylation and deacetylation of lysine residues can alter protein conformation and function, modulating several biological processes by influencing transcription regulation, enzyme activity, cell signaling, and protein-protein interactions^19^. Recent studies have suggested that sirtuins may play a role in virulence in bacteria and fungi. For example, sirtuins can modulate the expression of genes involved in virulence and pathogenesis in *Staphylococcus aureus*^53^ and *C. albicans*^54^.

In this study, we demonstrate the activity of four out of six *A. fumigatus* sirtuins and their role in this pathogenic fungus by combining phenotypic characterization of single knockout strains (Δ*sirA-E* and Δ*hstA*) and a strain displaying deletion of the six sirtuins (SIRTKO). SirE showed activity only on the histone H3-derived peptide, SirC was active only in the general peptide, and SirA and SirB were active on both peptides. All mutant strains constructed are viable under laboratory conditions, indicating that sirtuins are not essential genes. However, our results strongly suggest that sirtuins play roles in cell wall integrity, SMs production, and virulence. Notably, *A. fumigatus* Δ*sirE* and SIRTKO strains displayed a significant defect in radial growth, susceptibility to cell wall stressors, and attenuated virulence in both *G. mellonella* and mouse infection models. Interestingly, the wild-type virulence was not restored in the complemented strain (ΔsirE::sirE) on the murine model. This finding was similar to *Cryptococcus neoformans* sirtuin complement strains^55^, suggesting that loss of SirE activity resulted in a severe alteration in the acetylation code that cannot be completely restored.

Due to the hypovirulent phenotype of mutant strains, protein acetylation and gene expression profiles were investigated. We identified eight abundantly acetylated proteins encoded by genetic determinants of virulence in the SIRTKO mutant, which could represent direct or indirect targets of sirtuins. Moreover, hundreds of genes related to virulence were differentially expressed. Among upregulated genes, ketone metabolic process and secondary metabolism biosynthesis are enriched, while N-acetylglucosamine catabolic process and transmembrane transport were enriched among the downregulated genes.

The virulence of *A. fumigatus* is attributed to multiple factors^56^. The components of the cell wall, composed of layers of galactomannan, galactosaminogalactan, chitin, and beta-glucans contribute to the structural integrity of the cell wall and also play important roles in interactions with the host immune system^57,58^. Genes involved in cell wall biosynthesis are considered virulence factors and are frequently used as drug targets for treating fungal infections^59,60^. All constructed *A. fumigatus* sirtuin mutants showed some sensitivity to one of the tested cell wall stressors and differences in mycelial carbohydrate contents. In our transcriptome analysis, galactose biosynthesis and N-acetylglucosamine catabolic processes are enriched in the Δ*sirE* and SIRTKO strains, considering the downregulated genes, corroborating the phenotyping of these strains. Interestingly, UDP-galactopyranose mutase (*glfA)*^61,62^ an important virulence factor and a potential target for new antifungal compounds, was found abundantly acetylated in SIRTKO Kac-enriched proteome, suggesting a possible regulation by sirtuins. The loss of *sirE* in *A. fumigatus* increased the susceptibility to voriconazole and caspofungin, which target the synthesis of the main component of the fungal cell wall.

Filamentous fungi can produce a wide variety of SMs that contribute to their survival, fitness, pathogenicity, and virulence^11,63^. Histone acetylation status is an important regulator of chromatin structure, which impacts the expression of biosynthetic gene clusters in filamentous fungi. Sirtuins have been reported as regulators of SM production in *Aspergillus* spp^32,36,37,43,64,65^. The acetylome of *A. fumigatus* WT and SIRTKO strains demonstrate that the deletion of sirtuins promotes histone hyperacetylation and, therefore, could activate transcription of SMs gene clusters, resulting in a different metabolome profile, which was already seen in other fungi^66^. Combining the acetylome data with in vitro assays suggests that H3K56 could be the primary substrate for SirE, although it cannot be confirmed as the only substrate. The fact that *C. albicans* and *S. cerevisiae* have similar substrate preferences suggests that sirtuin function may be conserved across fungal species. Additionally, evidence indicates that *A. nidulans* SirE orthologue is nuclear^36^, reinforcing the idea that SirE’s main substrate might be histones. Our transcriptome data also reveal hundreds of differentially expressed genes related to secondary metabolism, corroborating the difference in the abundance of secondary metabolites observed in the mutant strain’s metabolome. Moreover, genes involved in various metabolic pathways and biological processes critical to the pathogenicity of *A. fumigatus*, such as SM biosynthesis, cell wall integrity pathway, cell signaling, an integral component of membrane, fatty acid metabolism, and pyruvate metabolism, were differentially expressed in the Δ*sirE* and SIRTKO strains.

In conclusion, we show that sirtuins are involved in several biological processes, probably by maintaining protein acetylation, which affects primary and secondary metabolism. The protein SirE is similar in sequence to the proteins Hst3/4 found in *S. cerevisiae* and *C. albicans*. SirE also deacetylates H3K56, which is a known substrate of Hst3/4 in *S. cerevisiae* and *C. albicans*^67,68^. In addition, the study found that knocking out SirE results in poor growth, similar to what is observed in yeast species. Moreover, SirE has a low identity with human sirtuins, making it a potential drug target. Interestingly, nicotinamide (NAM), a classical sirtuin inhibitor, exhibited significant antifungal activity against *C. albicans* and had a synergistic interaction with amphotericin B against *C. albicans* as well as other *Candida* spp. and *C. neoformans*^69,70^. Understanding the biology of these enzymes in *A. fumigatus* and how protein acetylation interferes with virulence may open perspectives for developing new molecules targeting protein acetylation. Further, in vivo studies will be necessary to assess the therapeutic potential of sirtuin inhibitors against the most common fungal pathogens.

## Methods

### In silico analysis

The protein sequences of *A. fumigatus* sirtuins were retrieved from the FungiDB database using the following gene IDs: Afu5g04120 (*hstA*); Afu4g12120 (*sirA*); Afu2g05900 (*sirB*); Afu6g09210 (*sirC*); Afu3g00520 (*sirD*); Afu1g10540 (*sirE*). The *S. cerevisiae* and human sirtuin sequences were obtained from the UniProt database^71^. The phylogenetic analyses were carried out using the Geneious Prime software with global alignment with free end gaps parameters, Blosum62 cost matrix, Jukes–Cantor genetic distance model and neighbor-Joining tree build method. Then, using the respective sequences of the sirtuins from *A. fumigatus* and *S. cerevisiae*, the predicted 3D structures were generated using the artificial intelligence algorithm Alphafold Protein Structure Database developed by DeepMind and EMBL-EBI^72,73^. PyMOL software was used for structural analyses and to generate the images. The amino acid identity analyses and phylogenetic tree were done using the software Geneious Prime.

### Protein expression and purification

The pET28a-AfSirA, pET28a-AfSirB, pET28a-AfSirC vectors were transformed into *E. coli* BL21 (DE3) and the pET28a-AfHstA, pET28a-AfSirD and pET28a-AfSirE vectors were transformed into *E. coli* Arctic Express by heat shock and selected in solid LB medium in the presence of 50 µg/mL ampicillin. For AfHstA, AfSirA, AfSirB, AfSirC, AfSirD and AfSirE protein expression, 10 mL of an overnight grown culture was inoculated into 500 mL of liquid LB medium in the presence of 50 µg/mL kanamycin and maintained for 2 h at 37 °C and 180 RPM. After 2 h of growth, 1 mM IPTG was added to the culture, and it was conditioned overnight at 12 °C and 180 RPM. The cultures obtained after induction of expression were centrifuged at 10,000 RPM for 15 min, resuspended with 10 mL of 20 mM Tris–HCl, pH 8.0, and centrifuged again. The bacterial precipitate was used in the protein purification steps.

Precipitates from the bacterial cultures obtained after induction were resuspended in lysis buffer (200 mM NaCl, 5% glycerol, 5 mM 2-beta mercaptoethanol, and 25 mM HEPES–NaOH, pH 7.5) and lysed using French Press apparatus. After centrifugation, the soluble fraction was used for the purification of heterologous proteins using Ni-NTA agarose resin (QIAGEN), previously equilibrated with equilibration buffer (200 mM NaCl, 5% glycerol, 5 mM 2-beta mercaptoethanol, 25 mM imidazole, 25 mM HEPES–NaOH, pH 7.5), and incubated for 1 h under stirring at 4 °C with the soluble fraction of proteins previously obtained by French Press. After incubation, the resin was washed 4× with 10 mL of wash buffer (200 mM NaCl, 5% glycerol, 5 mM 2-beta mercaptoethanol, 50 mM imidazole, 25 mM HEPES-NaOH pH 7.5). Proteins were eluted from the Ni-NTA column with 1 mL of elution solution (200 mM NaCl, 5% glycerol, 5 mM 2-beta mercaptoethanol, 250 mM imidazole, and 25 mM HEPES–NaOH, pH 7.5). The samples were analyzed using acrylamide gel electrophoresis to confirm the quality of purified proteins.

### Sirtuin deacetylase activity assay

Deacetylation assays were performed with purified recombinant AfHstA, AfSirA, AfSirB, AfSirC, AfSirD, and AfSirE proteins following the protocol established by Moretti et al.^74,75^. Briefly, the substrate for enzyme activity consists of a peptide containing an acetylated lysine residue associated with a fluorescent group and an eraser group (Abz-Gly-Proacetyl-Lys-Ser-Gln-EDDnp), where Abz is ortho-aminobenzoic acid; and EDDnp is N-[2,4-dinitrophenylethylenediamine], respectively. In addition, another substrate was tested with all proteins and was synthesized based on the histone site H3K56ac, following the same conditions cited (Abz-Tyr-Gln-Proacetyl-Lys-Ser-Thr-Gln-EDDnp). The reaction is divided into two steps, the first corresponding to the deacetylation reaction and the second corresponding to the trypsinization of the peptide. Reactions were performed in 96-well dark plate, incubated for 4 h at 37 °C in 50 μL containing sirtuin activity buffer (25 mM Tris–HCl pH 8; 137 mM NaCl; 2.7 mM KCl; 1 mM MgCl_2_), 0.6 mM NAD^+^ (Sigma Aldrich). After the reaction time, a 12 mM nicotinamide solution in 100 mM NaCl and 50 mM Tris–HCl, pH 8, containing 0.6 mM trypsin (Sigma Aldrich) was added to the reaction. After 30 min of incubation at 37 °C, fluorescence (Ex 320 and Em 420 nm) was measured using SpectraMax M3 equipment (Molecular Devices, Sunnyvale, CA, USA).

### Strain and culture conditions

The *A. fumigatus* strains used in this study (Table 1) have been maintained in complete medium (YG; glucose 2% (w/w), 0.5% yeast extract (w/w), 1X trace elements) or glucose minimal medium glucose (GMM; glucose 1% (w/w), 1x high nitrate salt solution (1.4 M NaNO_3_, 0.13 M KCl, 0.042 M MgSO_4_ ·7H_2_O and 0.22 M KH_2_PO_4_) and 1x trace elements (7.2 mM ZnSO_4_·7H_2_O, 17.7 mM H_3_BO_3_, 2.52 mM MnCl_2_·4H_2_O, 2.72 mM FeSO_4_·7H_2_O, 0.95 mM CoCl_2_·5H_2_O, 0.7 mM CuSO_4_·5H_2_O, 0.21 mM Na_2_MoO_4_·4H_2_O and 17.11 mM EDTA), pH 6.5) and (0.12% (w/v) uracil/uridine when required. For solid media, 1.5% agar (w/v) was added to YG or GMM, respectively. Plates were incubated at 37 °C for 36–72 h.

**Table 1:** Strains used in this study

*A. fumigatus* strains	Genotype	Reference
WT	*pyrG1*; ∆*KU80* (A1160-)	Da Silva et al.^96^
∆*sirA-*	*pyrG1*; ∆*KU80*; ∆*Afu4g12120*	This study
∆*sirB-*	*pyrG1*; ∆*KU80*; ∆*Afu2g05900*	This study
∆*sirC-*	*pyrG1*; ∆*KU80*; ∆*Afu6g09210*	This study
∆*sirD-*	*pyrG1*; ∆*KU80*; ∆*Afu3g00520*	This study
*∆sirE-*	*pyrG1*; ∆KU80; ∆*Afu1g10540*	This study
∆*hstA-*	*pyrG1*; ∆KU80; ∆*Afu5g04120*	This study
SIRTKO	*pyrG1*;∆*KU80*;∆*Afu4g12120*; ∆*Afu2g05900;∆Afu6g09210*; ∆*Afu3g00520*;∆*Afu1g10540*; *∆Afu5g04120*	This study
∆*sirA*^*+*^	∆*KU80*; ∆*Afu4g12120*; *pyrG1:*:*pyrG*	This study
∆*sirB*^*+*^	∆*KU80*; ∆*Afu2g05900; pyrG1*::*pyrG*	This study
∆*sirC*^*+*^	∆*KU80*; ∆*Afu6g09210*; *pyrG1*::*pyrG*	This study
∆*sirD*^*+*^	∆*KU80*; ∆*Afu3g00520*; *pyrG1*::*pyrG*	This study
∆*sirE*^*+*^	∆*KU80*; ∆*Afu1g10540*; pyrG1::pyrG	This study
∆*hstA*^*+*^	∆*KU80*; ∆*Afu5g04120*; pyrG1::pyrG	This study
SIRTKO^+^	∆*KU80*;∆*Afu4g12120*; ∆*Afu2g05900*;∆*Afu6g09210*; ∆*Afu3g00520*;∆*Afu1g10540*; ∆*Afu5g04120*; *pyrG1*::*pyrG*	This study
*sirE*^*H260Y*^	∆*KU80*; *Afu1g10540* H 260Y	This study
∆*sirE::sirE*	∆*KU80*; ∆*Afu1g10540*::*Afu1g10540*	This study

### CRISPR-Cas9 vector construction

The construction of the CRISPR-Cas9 vector for a target gene was performed as previously described^44,45^. Briefly, small DNA fragments (biobricks) were amplified by touchdown PCR using a PfuX7^76^ as polymerase and the vector pFC902 as a template. The primers and repair oligonucleotides were designed using the *A. fumigatus* genomic sequence of each target (*Afu4g12120; Afu2g05900; Afu6g09210; Afu3g00520; Afu1g10540* and *Afu5g04120*) and are listed in Supplementary Table 1. The PCR products were treated using DpnI (New England Biolabs) and purified using the Wizard SV Gel system and the PCR Clean-Up System (Promega).

The biobricks, which consist of a protospacer adjacent motif (PAM) sequence, were linked with a pFC330 (pyrG marker vector) by uracil-specific excision reagent (USER) fusion and USER cloning^77^. The mixture was used directly to transform thermo-competent *E. coli* DH5α cells. Transformants were plated on solid Luria Broth (LB) medium supplemented with 100 mg/ml ampicillin. Colonies were randomly selected for each construct and validated by colony PCR and PCR-product sequencing.

### Transformation in *A. fumigatus* and diagnostic PCR

Protoplast preparation of *A. fumigatus* was performed using protocols previously described^78^. For transformation, 8 µg of CRISPR vector and 10uL of 100 µM of repair oligonucleotide (90pb) were mixed with 100 µL protoplasts solution and 50 µL of PEG solution (25% (w/v)) in STC 50 (1.2 M sorbitol, 10 mM CaCl_2_ and 50 mM Tris–HCl pH 7.5). After 20 min on ice, 1 ml of PEG solution was added, and the mixture was incubated at room temperature for 20 min. Then, 3 mL STC 50 was added, and 1 ml of the suspension was poured onto a protoplast-recovery medium (supplemented with 1.2 M sorbitol). Plates were incubated at 37 °C until the appearance of isolate colonies. Monosporic purification was performed three times to obtain genetically pure mutants. Diagnostic PCR was performed using the genomic DNA of WT and mutant strains. The primers used are provided in Supplementary Table 1.

### Southern blot

Genomic DNA was extracted from mutant strains using phenol–chloroform. Briefly, ~80 µg of DNA was used for digestion with different restriction enzymes. The fragments were separated on 0.8% agarose gel and transferred by capillarity to the Amersham Hybond N+ membrane (GE Healthcare). DNA hybridization was performed with the Amersham AlkPhos Direct Hybridization Buffer, being detected using a multifunctional imaging system Typhoon FLA 9500 (GE Healthcare). Primers used for probe sequence are in Supplementary Table 2.

### Phenotype characterization

The sirtuin mutants were grown for 120 h at 37 °C in YG agar with cell wall stressors, such as Calcofluor White (CFW) and Congo Red (CR). The mutant strains were also cultivated in different temperatures (25, 37, and 45 °C) in GMM agar and subjected to antifungal susceptibility tests described below. The graphs of relative radial growth (treated/control) measurements were constructed by GraphPad Prism software. Significant statistical analysis was set using the Dunnett or ANOVA test and *p*-value < 0.05.

### Cell wall polysaccharides extraction and sugar quantification

All sirtuin mutant strains were incubated in MM for 36 h at 37 °C. After incubation, the mycelia were washed with distilled water and lyophilized. Fungal cell wall polysaccharides were extracted from 10 mg dry-frozen mycelia as described previously^79^. Briefly, 10 µL of extracted samples and standard sugars were subsequently analyzed by high-performance liquid chromatography (HPLC) on a Dionex Bio-LC system equipped with a CarboPac PA1 anion-exchange column and a CarboPac PA guard column by an isocratic elution of NaOH 16 mM. 1.0 mg/mL of fucose was added as an internal control.

### Minimal inhibitory concentration (MIC)

The MIC of sirtuin mutant strains was determined following the Clinical and Laboratory Standards Institute M38-A2 guidelines^80^. The susceptibility to azoles was compared to the reference strain (VRZ range 0.25–1.0 µg/mL: ITZ range 0.25–2.0 µg/mL and FLZ not susceptible). The MIC scores were obtained visually with the inoculums of 4.0 × 10^4^ conidia/mL after incubation at 37 °C for 48 h. The MIC was defined as the lowest concentration resulting in 100% growth inhibition compared to the drug-free control. The minimal effective concentration (MEC) of caspofungin was performed with GMM liquid media.

### Virulence assay in *Galleria mellonella* model

*G. mellonella* survival was assayed as previously described^81,82^. *G. mellonella* larvae were kept in sterile glass flasks with modified lids, with a hole in the center covered with an ultra-fine stainless steel wire mesh for better ventilation. Ten healthy *G. mellonella* larvae of similar weight (approximately 330 mg) were used in each group. Each larva was inoculated with 10 µL of conidia from the WT strain and the mutant strains of *A. fumigatus* on the last left proleg, directly on hemocoel. PBS was used as a control. The larvae were kept at 37 °C in the dark, and the mortality rate of larvae was monitored daily for 15 days; the larvae that did not present movement after touch stimulation were considered dead. Significant differences were observed by using a two-way ANOVA *p < 0.05.

### Virulence assay in immunosuppression of female BALB/c mice

Virulence assays were done in immunosuppressed female BALB/c mice. Wild-type BALB/c female mice, aged 6–8 weeks, were kept in the Animal Facility of the Laboratory of Molecular Biology of the School of Pharmaceutical Sciences of Ribeirão Preto, University of São Paulo (FCFRP/USP), in a clean and silent environment, under normal conditions of humidity and temperature, and with a 12 h light and dark cycle. The mice were given food and water ad libitum throughout the experiments. The procedures adopted in this study were performed following the principles of ethics in animal research and were approved by the Committee on Ethics in the Use of Animals (CEUA) of the FCFRP/USP (Permit Number: 08.1.1277.53.6; Studies on the interaction of *Aspergillus fumigatus* with animals) from the University of São Paulo, Campus of Ribeirão Preto. We have complied with all relevant ethical regulations for animal use.

Mice were immunosuppressed with cyclophosphamide (150 mg per kg of body weight), which was administered intraperitoneally on days −4, −1, and 2 prior to and post-infection. Hydrocortisone acetate (200 mg/kg body weight) was injected subcutaneously on day −3. *A. fumigatus* strains were grown on minimum medium (MM) for 2 days prior to infection. Fresh conidia were harvested in PBS and filtered through a Miracloth (Calbiochem). Conidial suspensions were spun for 5 min at 3000×*g*, washed three times with PBS, counted using a hemocytometer, and resuspended at 5.0 × 10^6^ conidia/mL. The viability of the administered inoculum was determined by incubating a serial dilution of the conidia on MM at 37 °C. Mice were anesthetized by halothane inhalation and infected by intranasal instillation of 1.0 × 10^5^ conidia in 20 ml of PBS. As a negative control, a group of mice received PBS only. Ten mice were used for each strain. Mice were weighed every 24 h from the day of infection and visually inspected twice daily. The statistical significance of comparative survival values was calculated by the Prism statistical analysis package by using Log-rank (Mantel–Cox) Test and Gehan–Brestow–Wilcoxon tests.

### Evaluation of clinical disease score

Mice were evaluated daily for weight change and clinical signs of aspergillosis. Each signal (piloerection, dyspnea, and ataxy) was scored 2, 3, or 4 points, respectively, and the sum of them per mouse corresponded to the daily clinical disease score. Mice that lost more than 5% and <10% of their weight in a 24 h period were assigned 1 point, and those with a loss superior to 10% received 2 points, which were added to the daily score. The score was zero in the absence of weight reduction or other aspergillosis signs. The quantification of the mice score was performed via a visual evaluation of the signs, in each mouse separately, daily, usually by at least two blinded examiners. Those mice that reached humane endpoints were euthanized to minimize mice suffering, and their cause of death was considered a fungal infection. Statistical analyses were performed using Graphpad Prism® 6 software. For all variables, normal distribution and homogeneous variance were tested. When the distribution was considered normal and with homogeneous variance, the parametric ANOVA test with Bonferroni’s post-test was used for three or more groups. Results were expressed as mean ± SEM (standard error of the mean). The differences observed were considered significant when *p* was <0.05.

### RNA extraction and RNA-Seq data analyses

10^6^ conidia/mL of WT, ∆*sirE*, and SIRTKO strains were cultured in 50 mL of MMG media at 37 °C for 36 h. The mycelia were collected, frozen in liquid nitrogen, and ground into powder. The RNA extraction was performed using Trizol followed by Direct-zol RNA Miniprep Kits (Zymo Research). The RNA quality was measured in a bioanalyzer, and samples with RIN > 7.5 were sequenced. The sequence data were submitted to.

### Quality control and alignment against the reference genome

RNA paired-end sequencing quality control was assessed through FastQC (www.bioinformatics.babraham.ac.uk/projects/fastqc) and multiQC^83^. An average of 45 million reads were sequenced per sample. Both adapters and low-quality bases (QV < 20) were trimmed from reads’ extremities using Trimmomatic^84^ with a minimum read length of 30 bp. All libraries were mapped against the “FungiDB-56_AfumigatusAf293_Genome.fasta” reference genome (retrieved from fungidb.org) through STAR aligner^85^ with default parameters. STAR-generated sorted BAM output files were used for assigning read counts to gene features with featureCounts^86^ with the following parameters: -s2 -p -M -O -fraction. We relied on the “FungiDB-56_AfumigatusAf293.gff” annotation file also downloaded from fungidb.org.

### Differential expression

The read counts table generated by featureCounts was then used as input for differential expression (DE) analyses relying on the EdgeR classic exact test (0.01 FDR threshold)^87^ DE pairwise comparisons were performed as WT-vs-Δ*sirE* and WT-vs-SIRTKO. The same read count table was also submitted to a multi-dimensional scaling (MDS) analysis using the *plotMDS* function from the EdgeR package. EnhancedVolcano (https://bioconductor.org/packages/release/bioc/html/EnhancedVolcano.html) was employed for an overall DE visualization through volcano plots. All tools described in this paragraph were run under the R environment version 4.1.0.

### Protein extraction

10^6^ conidia/mL of WT and SIRTKO *A. fumigatus* strains were cultivated in 50 mL of YPD (yeast peptone dextrose) for 36 h. Mycelia were frozen by liquid nitrogen and ground into a powder, followed by transfer to a 50 mL falcon tube and sonication five times on ice using a high-intensity ultrasonic processor in 5 mL of lysis buffer [8 M urea, 2 mM EDTA, 65 mM DTT, 30 mM nicotinamide, 3 μM trichostatin A, and 1 mM PMSF]. The remaining debris was removed by centrifugation at 10,000×*g* at 4 °C for 10 min. The protein was precipitated with cold 15% trichloroacetic acid for 2 h at 4 °C. After centrifugation at 10,000×*g* at 4 °C for 10 min, the supernatant was discarded, and the precipitate was washed three times with cold acetone. The protein was redissolved in a buffer [8 M urea and 100 mM NH_4_CO_3_ (pH 8.0)], and protein concentration was determined using a Bradford method^88^. For trypsin digestion, 5 mg of protein was reduced with 5 mM DTT for 25 min at 56 °C, alkylated with 14 mM iodoacetamide for 30 min at room temperature in the dark, and then diluted 1:5 with 50 mM NH_4_CO_3_. Trypsin was added at a 1:100 enzyme-to-protein mass ratio for overnight incubation at 37 °C and the next day topped up with the same amount following an extra 4 h incubation at 37 °C. Trypsin digestion was terminated by adding 0.1% TFA final concentration and desalted using Sep-Pak C18 500 mg sorbent cartridges (Waters). Peptides were dried by vacuum centrifugation. 10 µg were analyzed by LC–MS/MS for global proteome characterization, and the remaining were reserved for the enrichment of lysine-acetylated peptides.

### Affinity enrichment of lysine-acetylated peptides

PTMScan® Acetyl-Lysine Motif [Ac-K] Kit (CSL) was used to enrich 5 mg of protein per sample according to the manufacturers and Schilling, B*.* et al.^89^ protocols, followed by LC–MS/MS analysis. Briefly, peptide samples resuspended in IAP buffer were incubated with 100 μL of antibody-bead conjugates specific for acetyl-lysine (Cell Signaling Technologies, PTMScan kits #13416). Immunoprecipitation proceeded overnight at 4 °C with gentle mixing. Next, beads were washed twice with 1 mL ice-cold IAP buffer, and thrice with 1 mL ice-cold IAP buffer. Bound peptides were eluted sequentially with 45 mL and then 55 mL of 0.15% TFA for 5 min each at RT. Eluted PTM peptides were then directly loaded onto C18 Stage Tips (Waters), desalted with 0.2% FA in water, and eluted with a solution containing 50% ACN, 49.8% water, and 0.2% FA. Eluted peptides were dried completely and stored at −80 °C until further analysis.

### LC–MS/MS

The peptide mixtures of both total proteome and Kac-enriched fraction were analyzed using the EASY-nLC II (Proxeon Biosystems) equipped with the analytical column PicoFrit C18 (20 cm × 75 μm i.d., 5 μm; New Objective) at a constant flow rate of 300 nl/min coupled to the LTQ-Orbitrap Velos mass spectrometer (Thermo Fisher Scientific). Peptides were resolved over a 120 min gradient, 2–30% B (solvent A—water with 0.1% formic acid; solvent B—acetonitrile with 0.1% formic acid). Eluting peptides were analyzed by the mass spectrometer operating in a positive polarity and Top5 data-dependent acquisition mode. Precursor ions (*m*/z 300–1600) were scanned in the Orbitrap with a resolution defined to *r* = 60,000 and 1E6 target ions. Up to 5 most intense multiply charged ions (8E4 target ions) were isolated through a 2.1-Da window and activated by higher-energy collisional dissociation (HCD), with a normalized collision energy of 40%. Product ions were scanned by the Orbitrap with a resolution defined as *r* = 7500. Dynamic exclusion was enabled with an exclusion list of up to 400 ions, an exclusion duration of 30 s, and a count repetition of 2.

### Proteomics data analysis

Mass spectra were processed using MaxQuant 2.0.3.0 for database search and label-free quantification of protein groups and Kac-sites-containing peptides. Thermo.raw files of both *A. fumigatus* proteome and Kac-enriched samples were loaded and analyzed in parallel as unmatching fractions, using specific search parameters for the proteome and Kac-enriched fraction as described below. Database search was performed against Af293 FungiDB protein base (release 51, 9840 sequences. 4,866,332 residues). Enzyme specificity was set to trypsin/P (P1 = K and R), allowing up to 2 or 3 missed cleavage sites for proteome or Kac-enriched samples, respectively. Cysteine carbamidomethylation (+57.02 Da) was set fixed modification and oxidation of methionine (+15.99 Da), N-terminus acetylation (+42.01 Da) and lysine acetylation (+42.01 Da; only for Kac-enriched samples) were set variable modification. Mass error tolerances were set to 10 ppm for precursors and 0.02 Da for product ions. PSM and protein hits with at least 1 unique peptide were filtered at a 1% FDR threshold calculated using reversed-decoy sequences. Minimum and delta scores for modified peptides were set to 40 and 6, respectively.

MaxLFQ^90^ was enabled for label-free quantification (LFQ) based on precursor mass peak (MS1) intensities. Match between runs within the same sample types (i.e. proteome or Kac-enriched fraction) was enabled. Label-free quantification of protein groups identified in the proteome fraction was derived from the intensities of unique plus razor peptides assigned to each protein group, including peptides containing oxidized methionine and N-terminally acetylated peptides mapped to protein N-termini. Neither Kac-acetylated peptides nor their unmodified counterparts were considered for label-free quantification of protein groups. The abundances of Kac-containing peptides were estimated based on the MS1 intensities of precursor ions assigned to each Lysine-acetylation site identified. Site occupancy was automatically calculated for the cases where both modified and unmodified peptides were detected for a protein group.

Relative quantification between conditions was performed at protein and peptide levels for the proteome and Kac-enriched fraction using Perseus v1.6.15^91^. Only proteins and Kac-sites quantified in all three replicates of at least one sample group were considered for statistical analysis. Kac-sites missing quantitative information in the second group were subject to Perseus’ Gaussian model for zero replacement to avoid comparing valid group means against zeros. Group differences were tested by Student’s *t*-test with permutation-based FDR correction for multiple testing, with a significance threshold of *q*-value ≤ 0.05 and S0 = 0.1.

### SMs extraction and UHPLC-HRMS/MS analysis

The mutant strains (10^5^ spores) were cultivated in solid GMM media for 120 h at 37 °C. After culturing, the total content from each Petri dish was cut into small pieces (2 × 2 cm), and metabolites were extracted with equal quantities of methanol (MeOH) by ultrasonic extraction at room temperature for 1 h. Supernatant was then vacuum-filtered, and the solvent was removed under reduced pressure. The same procedure was performed for the control culture medium. Final extracts were stored at −20 °C. For sample preparation, 1 mL of HPLC grade MeOH was added to each extract and sonicated until complete dissolution. 500 µL of the obtained extracts were transferred to vials and diluted with HPLC grade MeOH to a total volume of 1 mL. Quality control (QC) samples were prepared with 55 µL of each sample and diluted to a final volume of 1 mL with HPLC grade MeOH. UHPLC-HRMS/MS positive mode analysis was performed in a Thermo Scientific QExactive Hybrid Quadrupole-Orbitrap Mass Spectrometer. As a stationary phase, a Thermo Scientific column Accucore C18 2.6 µm (2.1 mm × 100 mm × 1.7 µm) was used. Mobile phase was 0.1% formic acid (A) and acetonitrile (B). Eluent profile (A/B %): 95/5 up to 2/98 within 10 min, maintaining 2/98 for 5 min, down to 95/5 within 1.2 min and maintaining for 8.8 min. The total run time was 25 min for each run, and the flow rate was 0.3 mL min^−1^. The injection volume was 6 µL. MS spectra were acquired with *m/*z ranges from 100 to 1500, with 70,000 mass resolution at 200 Da, 1 microscan, and an AGC target of 16 with a maximum ion injection time set to 100 ms. Ionization parameters: sheath gas flow rate (45), aux gas flow rate (10), sweep gas flow rate (2), spray voltage (3.5 kV), capillary temperature (250 °C), S-lens RF level (50) e auxiliary gas heater temperature (400 °C). MS^2^ spectra were collected using a normalized collision energy of 30 eV, and the 5 most intense precursors per cycle were selected and measured with 17,500 mass resolution at 200 Da, 1 microscan, and an AGC target of 1^5^ with a maximum ion injection time for MS/MS scans set to 50 ms.

### UHPLC-HRMS/MS data processing and Feature-Based Molecular Networking (FBMN)

Raw UHPLC-HRMS/MS data were converted into mzML format files in MSConvert with 64-bit binary encoding precision and peak peaking^92^. Feature detection was performed in MZmine2 (v.2.53). Raw data, converted mzML data, MZmine2 batch queue describing data processing steps and parameters, MZmine2 output, and sample metadata files were deposited into the MassIVE repository (accession number: MSV000087365). Multivariate analyses were performed with the MetaboAnalyst tool (v.4.0)^93^. Briefly, the features list was filtered if their relative standard deviation was>25% in QC samples, followed by normalization by sum and Pareto scaling. Principal component analysis (PCA) was used to evaluate the robustness of data acquisition and to explore the differences in the chemical space of each strain. For FBMN the data were filtered by removing all MS/MS fragment ions within ±17 Da of the precursor m/z. MS/MS spectra were window-filtered by choosing only the top 4 fragment ions in the ±50 Da window throughout the spectrum. The precursor and fragment ion mass tolerance were both set to 0.02 Da. A molecular network was then created where edges were filtered to have a cosine score above 0.65 and more than 4 matched peaks. Furthermore, edges between two nodes were kept in the network only if each of the nodes appeared in each other’s respective top 10 most similar nodes. Finally, the maximum size of a molecular family was set to 100, and the lowest-scoring edges were removed from molecular families until the molecular family size was below this threshold. The spectra in the network were searched against GNPS spectral libraries^94^ and then were filtered in the same manner as the input data. All matches between network and library spectra were required to have a score above 0.65 and at least 4 matched peaks. The FBMN GNPS job is available online (https://gnps.ucsd.edu/ProteoSAFe/status.jsp?task=058516efad8f4af081b763c52404a7f7). The resulting networks were displayed and analyzed with Cytoscape (v.3.8.2)^95^.

### Statistics and reproducibility

Data are expressed as mean ± standard error of at least three independent experiments. *P* values were calculated using Prism (GraphPad Inc.). The statistical significance of the differences was determined using the tests shown in the figure legends. Statistical significance was set at *P*  <  0.05. Sample size and numbers are indicated in each figure legend.

### Supplementary information

Supplementary Information

Description of Additional Supplementary Files

Supplementary Data 1

Supplementary Data 2

Supplementary Data 3

Supplementary Data 4

Supplementary Data 5

## Data Availability

*Proteomic data*: the mass spectrometry proteomics data have been deposited to the ProteomeXchange Consortium via the PRIDE partner repository with the dataset identifier PXD044964 and 10.6019/PXD044964. *Metabolomic data*: Metabolome data were deposited into the MassIVE database under accession number MSV000087365 and are available at the following https://massive.ucsd.edu/ProteoSAFe/dataset.jsp?task=90955786692d4cff950a1bcd516e922a. *Transcriptomic data*: RNA-Seq data were deposited into the NCBI-Bioproject database under accession number PRJNA1029295 and are available at the following https://www.ncbi.nlm.nih.gov/bioproject/?term=PRJNA1029295. The source data behind the graphs in the paper can be found in Supplementary Data 5. Uncropped and unedited images for the gels/blots are available in Supplementary Fig. 9. All other data are available from the corresponding author on reasonable request.
