# Supplementary material for: Sirtuin E deacetylase is required for full virulence of Aspergillus fumigatus
Source: Commun Biol. 2024 Jun 8;7:704. doi: 10.1038/s42003-024-06383-3 (PMC11162503; doi:10.1038/s42003-024-06383-3)
Supplement: Supplementary file 1 — Supplementary Information [file 42003_2024_6383_MOESM1_ESM.docx]

***Sirtuin E deacetylase is required for full virulence of Aspergillus fumigatus***

Natália S. Wassano, Gabriela B. da Silva, Artur H. Reis, Jaqueline A. Gerhardt, Everton P. Antoniel, Daniel Akiyama, Caroline P. Rezende, Leandro X. Neves, Elton Vasconcelos, Fernanda L. de Figueiredo, Fausto Almeida, Patrícia A. de Castro, Camila F. Pinzan, Gustavo H. Goldman, Adriana F. P. Leme, Taicia P. Fill, Nilmar S. Moretti, André Damasio

**SUPPLEMENTARY INFORMATION**

**Suplementary Table 1.** Primers and oligonucleotides used to build the biobricks for vector construction and transformation.

| **CRISPR-Cas9** | **Name (IDT)** | **Sequence** | **PCR confirmation primers** |
| --- | --- | --- | --- |
| *sirA* AFU4G12120 | NW_AFU4G12120_P1Fwd | AGAACCAAAUTTACACGTTTTAGAGCTAGAAATAGCAAG | GACGACCACCCAAGACTGTT |
|  | NW_AFU4G12120_P1Rev | ATTTGGTTCUGGGTTGCATCATCCGTGAATCGAAC | CGGCCTTTGATTCTGGATAG |
|  | NW_AFU4G12120_P2Fwd | ATCGGTTAGGGAUGGCGCGTTTTAGAGCTAGAAATAGCAAG |  |
|  | NW_AFU4G12120_P2Rev | ATCCCTAACCGAUAGTGCATCATCCGTGAATCGAAC |  |
|  | OLIGO_AFU4G12120 | GTCATCTCCGACCGCTCTACCCAGAGATACCCAGAACCAAATTTACGCAGGTCTGTGGGTTTATTTTAATATTCTTGCAGTATCATCTGT |  |
| *sirB* AFU2G05900 | NW_AFU2G05900_P1Fwd | ACACACCAGCAUAGGTTTTAGAGCTAGAAATAGCAAG | CGCAATGCGCTTTTATCTTG |
|  | NW_AFU2G05900_P1Rev | ATGCTGGTGTGUGCAAGTTGCATCATCCGTGAATCGAAC | ACTCCCTCCATGTCTATCCAGA |
|  | NW_AFU2G05900_P2Fwd | AATGAAGAUGAGCTAGTTTTAGAGCTAGAAATAGCAAG |  |
|  | NW_AFU2G05900_P2Rev | ATCTTCATUAACTCTGCATCATCCGTGAATCGAAC |  |
|  | OLIGO_AFU2G05900 | TTTACCAGCTGCATTGACCGCCGACGAAACTTGCACACACCAGCAGAGCTATGGCCATGAAAAGACACCAGAGGTAGCCCAGGGGTATAC |  |
| *sirC* AFU6G09210 | NW_AFU6G09210_P1Rev | AGCACCAAUGACGTGCATCATCCGTGAATCGAAC | TCTCTCAGACAGCGAGCGTA |
|  | NW_AFU6G09210_P1Fwd | ATTGGTGCUCCGCATTGTTTTAGAGCTAGAAATAGCAAG | AGCAGGCTAGGTGCTCATGT |
|  | NW_AFU6G09210_P2Rev | ACTCTTCGCUAAACTGCATCATCCGTGAATCGAAC |  |
|  | NW_AFU6G09210_P2Fwd | AGCGAAGAGUGTGCAGGTTTTAGAGCTAGAAATAGCAAG |  |
|  | OLIGO_AFU6G09210 | ACTTGGAGTCAATCTTTTTGCTACCCCACGTCATTGGTGCTCCGCGTGCAGAGGCAAAGCTACATGTTTTAACCTTTGAATTCCTGAAGA |  |
| *sirD* AFU3G00520 | NW_AFU3G00520_P1Rev | AACCCCTCUGATACCTGCATCATCCGTGAATCGAAC | TGTCGCAGTGAGATGAGGTC |
|  | NW_AFU3G00520_P1Fwd | AGAGGGGTUGGAATGTTTTAGAGCTAGAAATAGCAAG | CGGAATGCGAAAGAGAAGTC |
|  | NW_AFU3G00520_P2Rev | AGATCATAUCTACTGCATCATCCGTGAATCGAAC |  |
|  | NW_AFU3G00520_P2Fwd | ATATGATCUACATGCAGTTTTAGAGCTAGAAATAGCAAG |  |
|  | OLIGO_AFU3G00520 | AGACCTATATTCACCCGACCATCCACGAGGTATCAGAGGGGTTGGTGCATGGAAAATGGGCTTACTGGTTCCGTCGTTTAAAATGAGTCA |  |
| *sirE* AFU1G10540 | NW_AFU1G10540_P1Rev | ACCTAAGCCAUAGGAGGATGCATCATCCGTGAATCGAAC | GAACGATGGATCCTGATGCT |
|  | NW_AFU1G10540_P1Fwd | ATGGCTTAGGUTGGTTTTAGAGCTAGAAATAGCAAG | CAAAATAAACACAGCACATCG |
|  | NW_AFU1G10540_P2Rev | ACGTCCATCCAUCGATGCATCATCCGTGAATCGAAC |  |
|  | NW_AFU1G10540_P2Fwd | ATGGATGGACGUGCGGCGTTTTAGAGCTAGAAATAGCAAG |  |
|  | OLIGO_AFU1G10540 | TCCTTCCACCACAACACCACCCACTTCTTCCTCCTATGGCTTAGGCGGCTGGATTATCCTGTAGTTCTTGCTGTATACTCTACGGGATCA |  |
| *hstA* AFU5G04120 | NW_AFU5G04120_P1Rev | ACGAAATACCUTTATTGCATCATCCGTGAATCGAAC | GGTGATCTGATCGCAACTTGT |
|  | NW_AFU5G04120_P1Fwd | AGGTATTTCGUGGCTGGTTTTAGAGCTAGAAATAGCAAG | ATCATATCCACGGTCTACCA |
|  | NW_AFU5G04120_P2Rev | ATGTACAGTGUACAGCGTGCATCATCCGTGAATCGAAC |  |
|  | NW_AFU5G04120_P2Fwd | ACACTGTACAUTTTGTTTTAGAGCTAGAAATAGCAAG |  |
|  | OLIGO_AFU5G04120 | ATCTTACATCATATCCACGGTCTACCATAATAAAGGTATTTCGTGATTTTCGGATTTAAGTCCATATATGCATAGACTGTTAGATAGTTA |  |
| Vector | CSN438-Fwd | GGGTTTAAU GATCACATAGATGCTCGGTTGACA |  |
|  | CSN790-Rev | GGTCTTAAU ACCCTGAGAAGATAGATGTGAATGTG |  |
| Annealing sequencing tRNA, annealing sequencing sgRNA, protospacer, overhangs to cassette PacI/Nt.BbvCI, substitution T-> U | | |  |

**Suplementary Table 2**. Primers used for probe sequence in Southern blot assays.

| **Southern Blot** | **Name** | **Sequence** | **Digesting enzymes** |
| --- | --- | --- | --- |
| *sirA* | SB_Af12120_Fwd | TTGGCGGAAAAGAAGAAGAA | SacI |
|  | SB_Af12120_Rev | CCAGGTCGTAGAGGGTCAAA |  |
| *sirB* | SB_Af00590_Fwd | GAACACGTCAATTGGGAGGT | SacI and NcoI |
|  | SB_Af00590_Rev | GAGGCACAACGATGTAGCAA |  |
| *sirD* | SB_Af00520_Fwd | TTGCGATATGCCACATCAGT | SalI and EcoRI |
|  | SB_Af00520_Rev | GACCTCATCTCACTGCGACA |  |
| *sirE* | SB_Af10540_Fwd | GACCAATGAGACTGCAGCAA | BglII |
|  | SB_Af10540_Rev | CACGGTGGAAAGGACTCAAT |  |
| *hstA* | SB_Af04120_Fwd | TTCAACCGCCAGTGTCATTA | EcoRI |
|  | SB_Af04120_Rev | CCGGGTTCTTGAGAAAATCA |  |
| *sirC* | SB_Af09210_Fwd | ATCGCTGCGAGTAGGGTAGA | XbaI and Xhol |
|  | SB_Af09210_Rev | AAAAGTCGTACGCTCGCTGT |  |

**
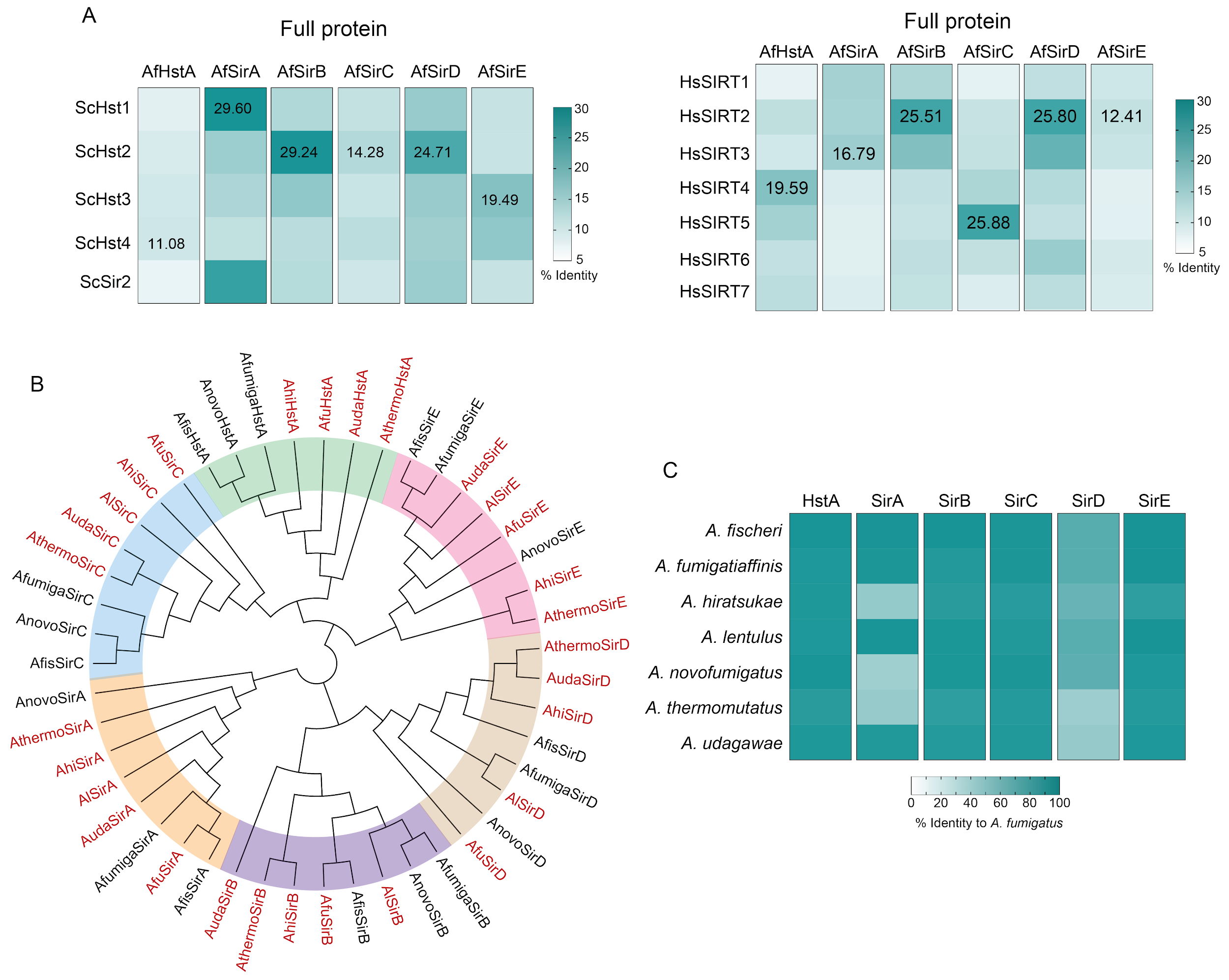
**

**Suplementary Figure 1.** *A. fumigatus* sirtuins compared to other species. **A)** Amino acid identity of full-length *A. fumigatus* sirtuins compared to *S. cerevisiae* (left) and human (right) proteins. **B)** Phylogenetic tree of sirtuins from *Aspergillus* spp. Afu: *A. fumigatus*; Afi: *A. fischeri*; Afu: *A. fumigatiaffinis*; Auda: *A. udagawae*; Al: *A. lentulus*; Anovo: *A. novofumigatus*; Ahi: *A. hiratsukae*; Athermo: *A. thermomutatus*. Sirtuins from opportunistic human pathogens are displayed in red. **C)** Full amino acid sequence identity of human pathogenic *Aspergillus* species compared to *A. fumigatus*.

**
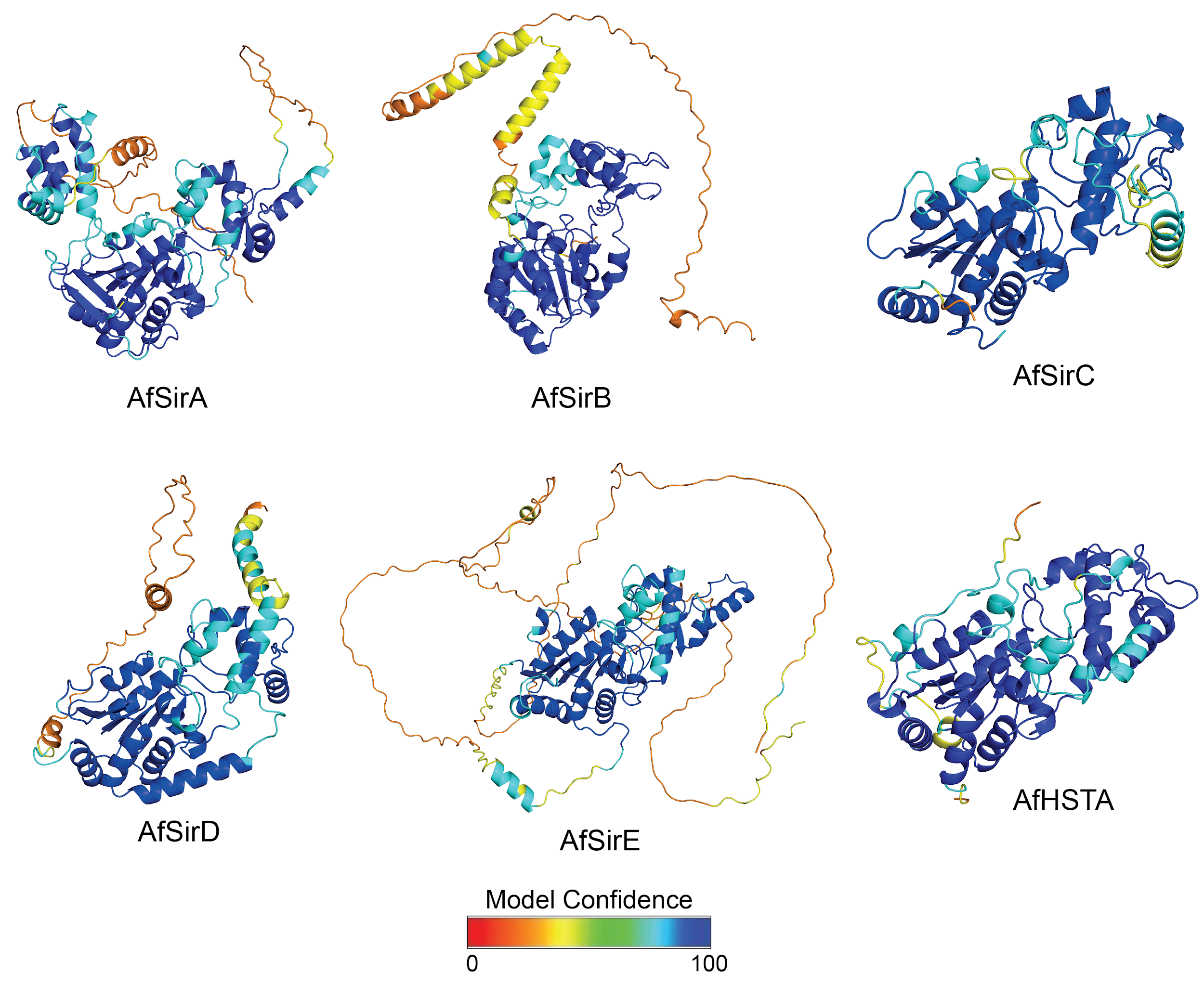
**

**Suplementary Figure 2. 3D predicted model of *A. fumigatus* sirtuins. All** models were generated using the AlphaFold tool based on the amino acid sequences deposited in the FungiDB (<https://fungidb.org/fungidb/app>). Dark/light blue colors indicate highly confident structural regions.


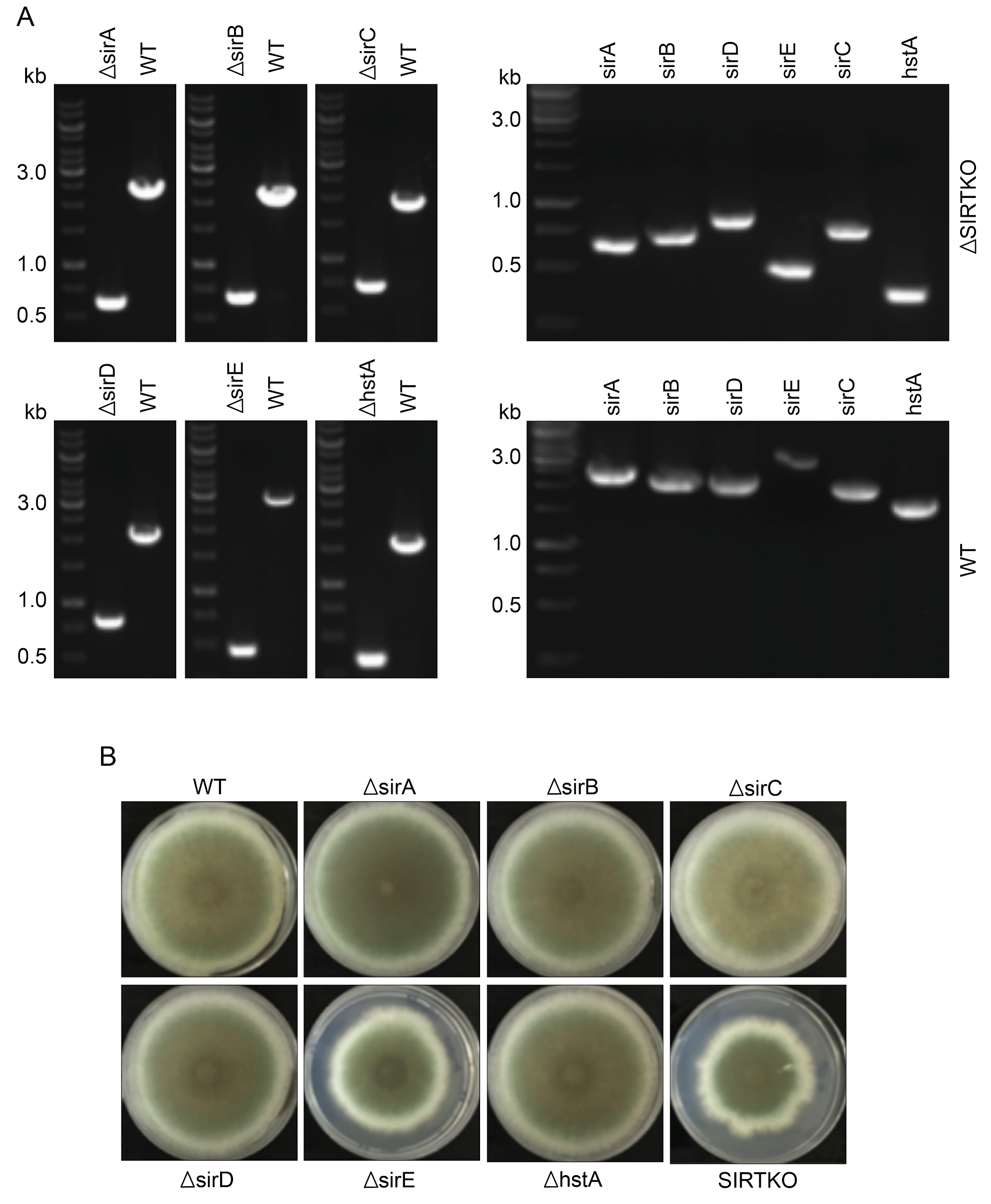


**Suplementary Figure 3. Validation of *A. fumigatus* mutant strains.** **A)** Fungal growth in Glucose Minimal Media (GMM) for 120h at 37°C. **B)** Gene deletions were validated by diagnostic PCR, confirming the deletion of the six sirtuin genes. L= ladder; Δ= mutant; WT= Wild Type.

**
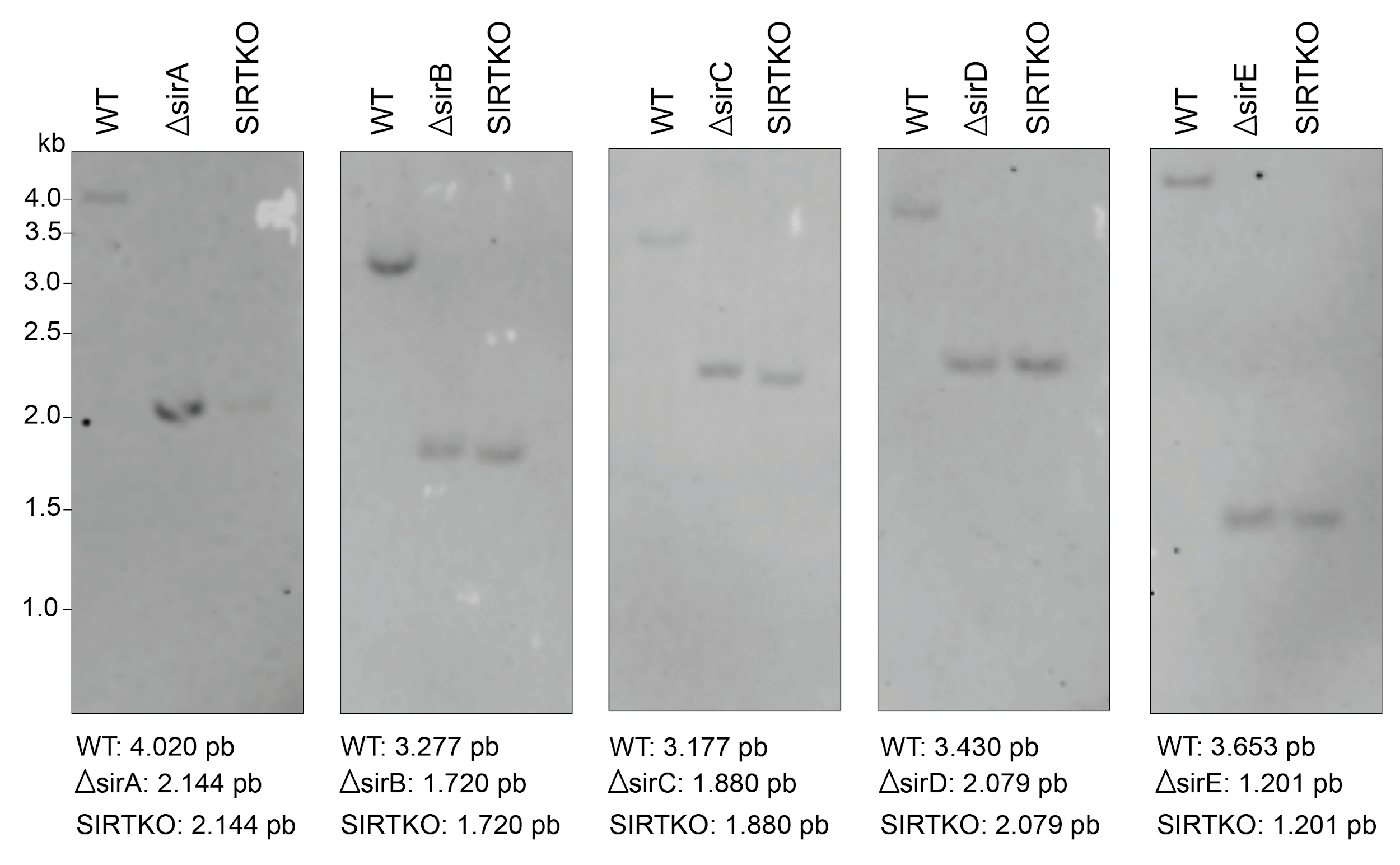
**

**Suplementary Figure 4. Southern blot analysis of *A. fumigatus* mutant strains.** The expected fragment lengths in the single mutants and SIRTKO are detailed at the bottom of the image. Primers and digesting enzymes are listed in Supplementary Table 2.

**
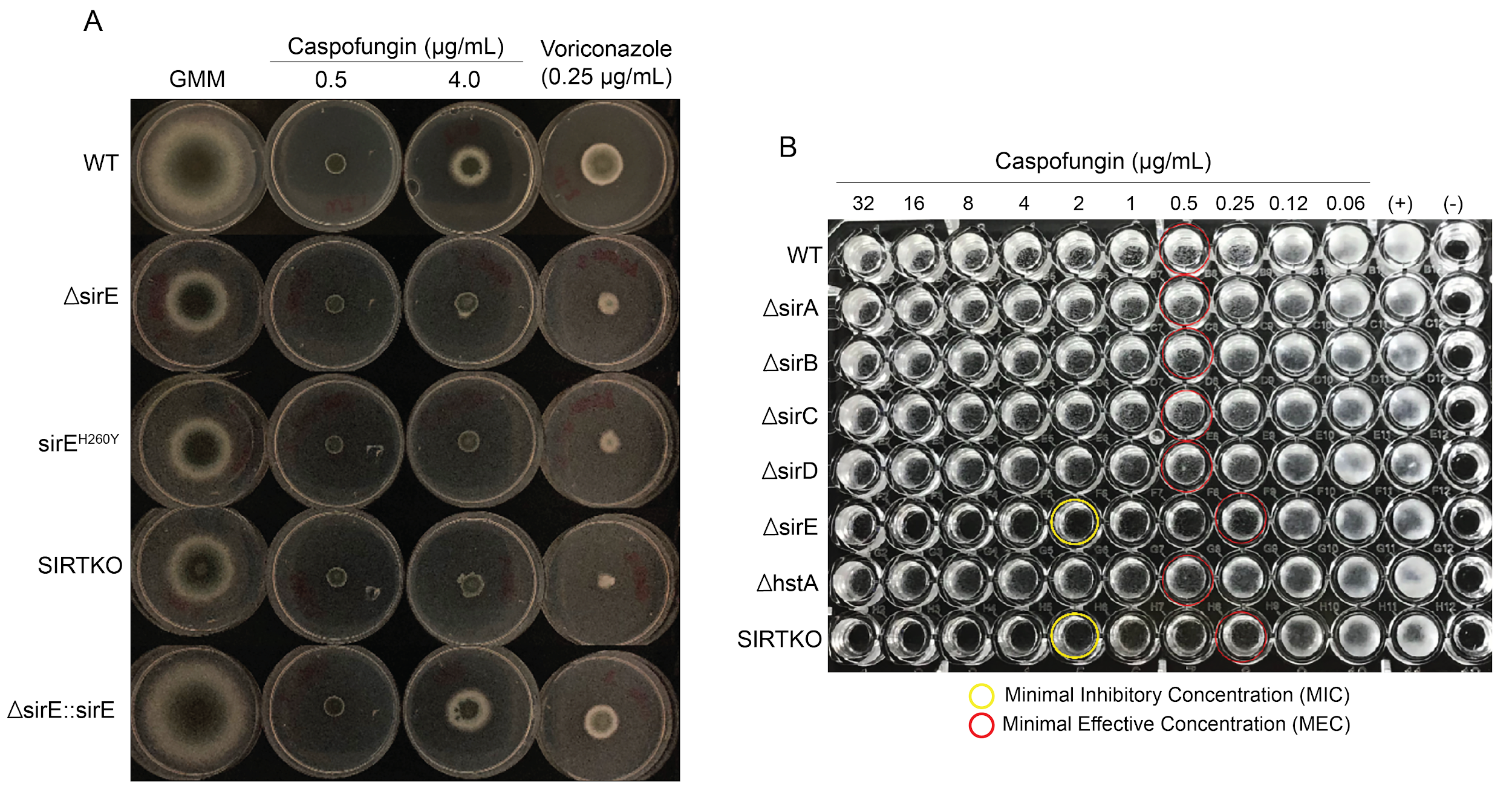
**

**Suplementary Figure 5. Antifungal susceptibility of *A. fumigatus* mutant strains**. **A)** *A. fumigatus* A1160 (WT), sirtuin E mutant strains (∆*sirE*, *sirE*^H260Y^) and complemented strain (∆sirE::sirE) grown on Glucose Minimal Media supplemented 0.12% (w/v) uracil/uridine and with 0.5 and 4.0 µg/ml of caspofungin and 0.25 µg/ml voriconazole. **B)** Caspofungin Minimal Effective Concentration (MEC) based on CLSI protocol supplemented 0.12% (w/v) uracil/uridine. (+) positive control and (-) negative control.


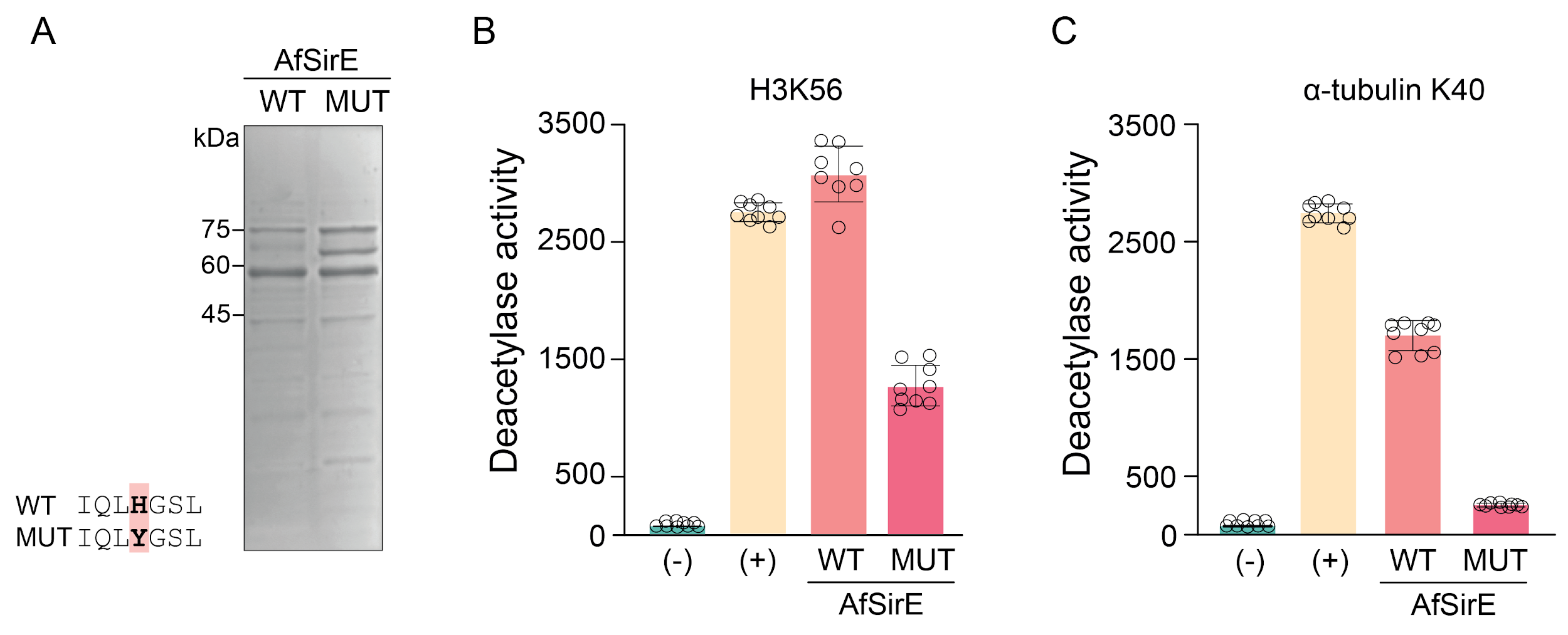


**Suplementary Figure 6. In vitro deacetylation activity of AfSirE^H260Y^ heterologous protein. A)** SDS-PAGE of AfSirE and AfSirE^H260Y^ purified proteins used in the activity assays. **B-C)** Deacetylation assay using H3K56ac and a-tubulin K40ac peptide showing the reduction in the activity of AfSirE^H260Y^ (MUT) compared to AfSirE (WT). (-): negative control; (+): positive control.


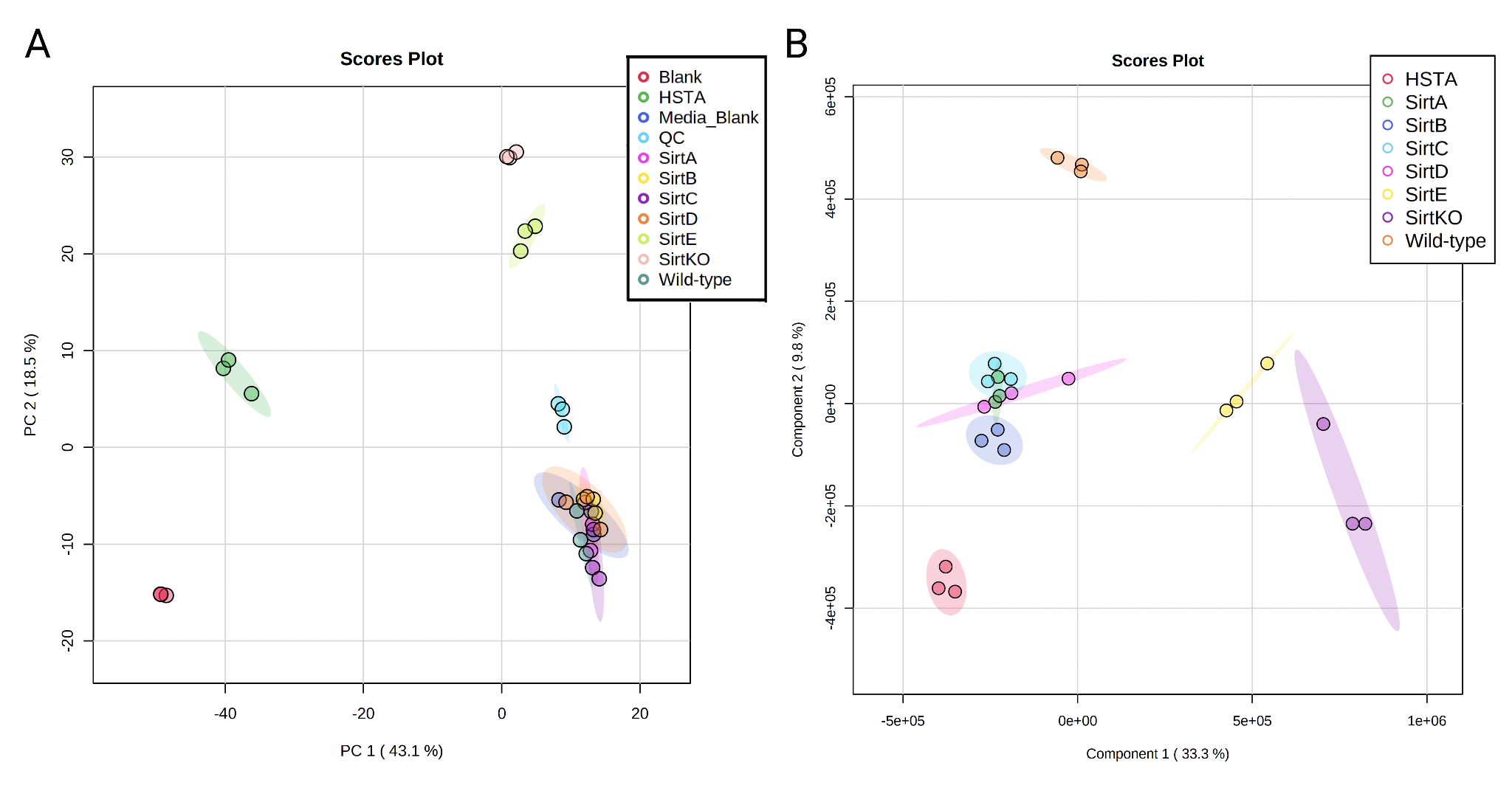


**Suplementary Figure 7.** Samples distribution in the metabolome assays for secondary metabolites identification. **A**) Principal component analysis (PCA) of the methanolic extracts of *A. fumigatus* sirtuin mutant strains. The PCA model was built with the extracts of all biological groups, analytical blanks, QC samples, and GMM media. **B)** Partial Least-squares Discriminant analysis (PLS-Da). The assay was performed in three replicates (n=3).


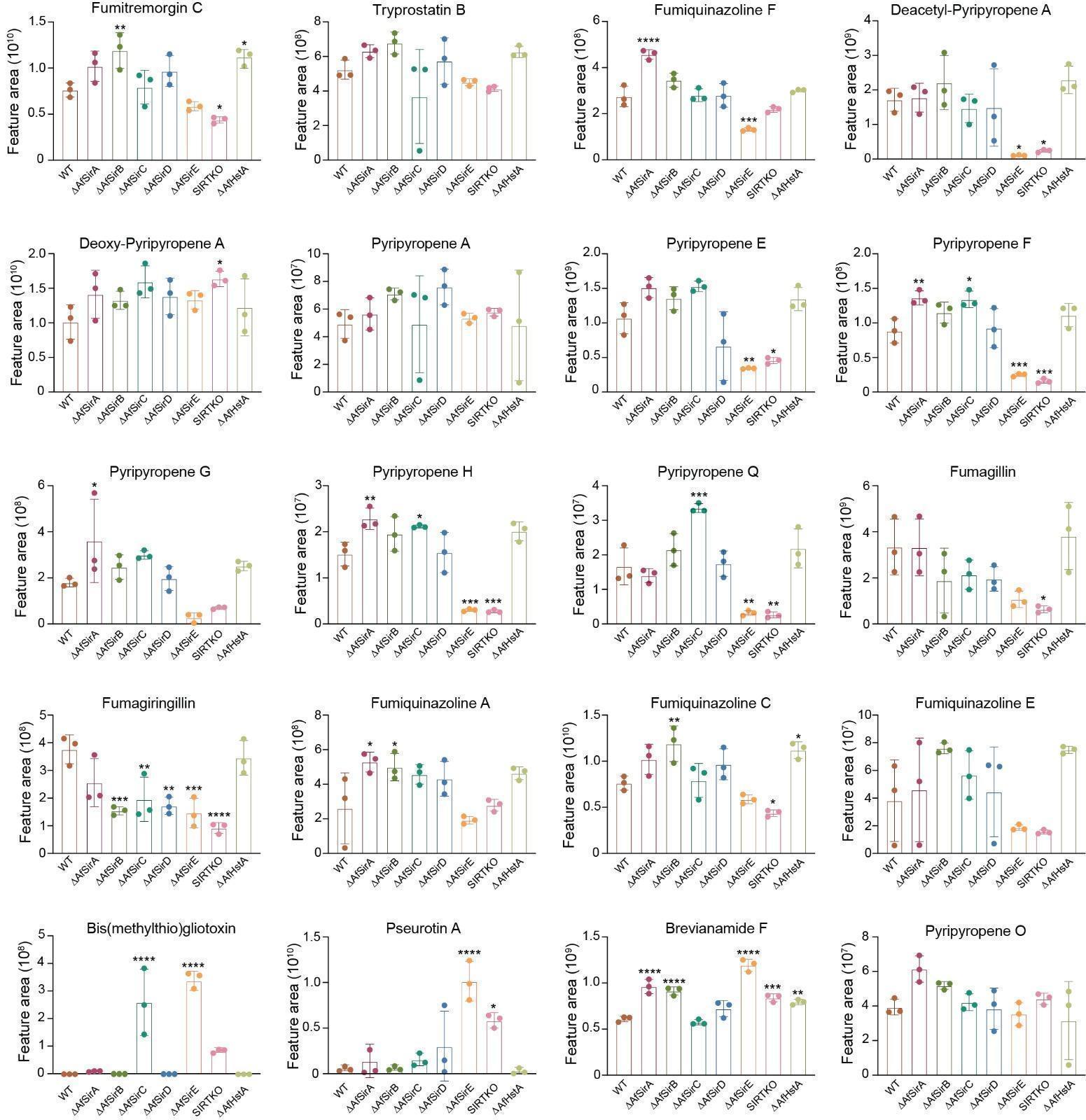


**Suplementary Figure 8.** Relative quantification of putative secondary metabolites in *A. fumigatus* sirtuin mutant strains. The data represent the average value of three replicates. The error bars represent the standard deviation, p ≤ 0.05.


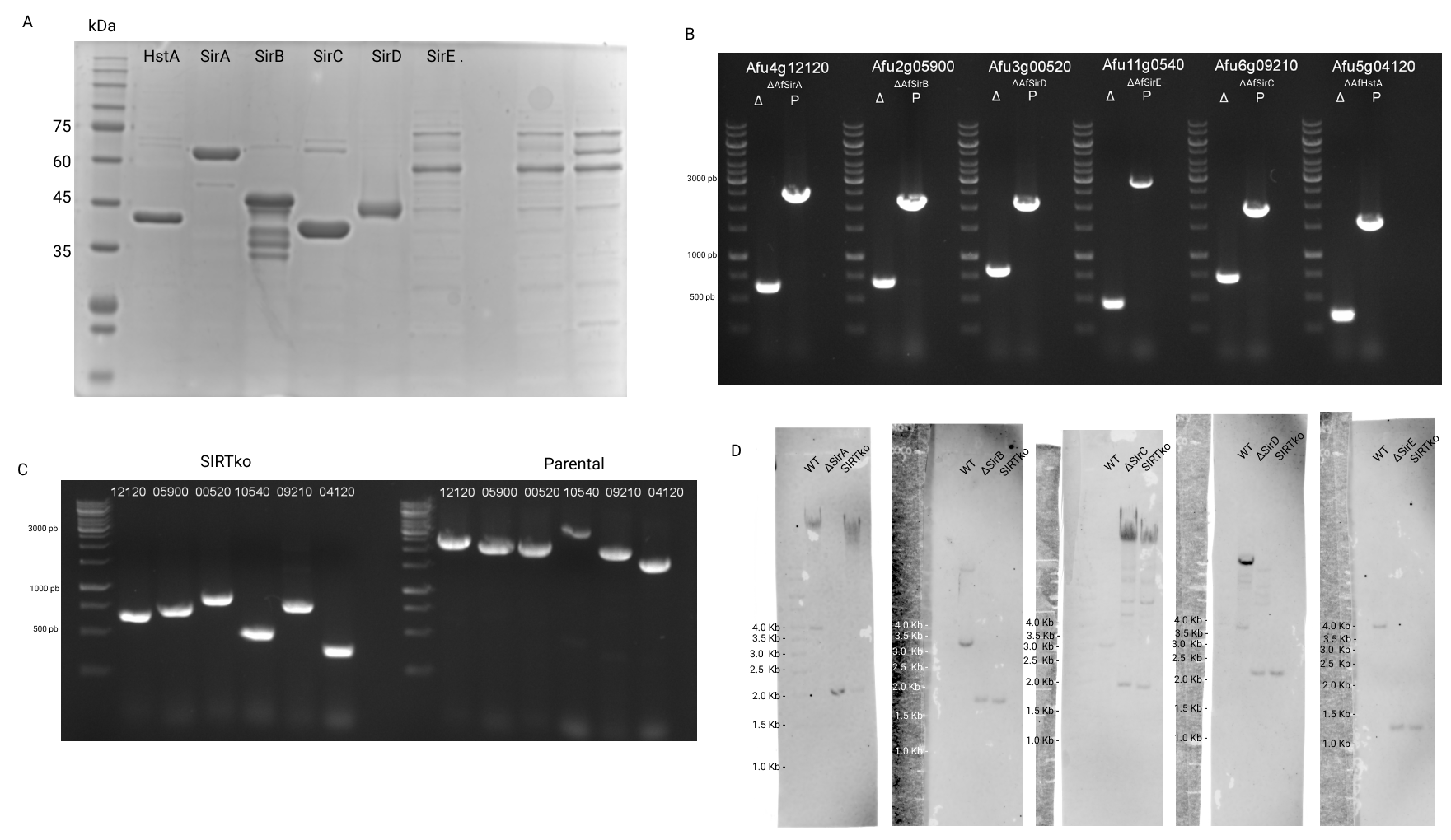


**Supplementary Figure 9. Uncropped and unedited images for the gels/blots. A)** Figure 1e and Supplementary Figure 6a; **B and C)** Supplementary Figure 3; **D)** Supplementary Figure 4.
