# Supplementary material for: Sirtuin E deacetylase is required for full virulence of Aspergillus fumigatus
Source: Commun Biol. 2024 Jun 8;7:704. doi: 10.1038/s42003-024-06383-3 (PMC11162503; doi:10.1038/s42003-024-06383-3)
Supplement: Supplementary file 2 — Description of Additional Supplementary Files [file 42003_2024_6383_MOESM2_ESM.pdf]

Description of Additional Supplementary Files

File name: Supplementary Data 1.

Description: Total proteome of SIRTKO and ΔsirE strains.

File name: Supplementary Data 2
Description: Acetylome analysis of Kac enriched fraction of SIRTKO and ΔsirE strains.

File name: Supplementary Data 3

Description: RNAseq analysis of DEGs (WT vs. SIRTKO, WT vs. ΔsirE, and SIRTKO vs. ΔsirE).

File name: Supplementary Data 4

Description: Annotation and identification of enriched pathways using the differentially expressed genes in SIRTKO and ΔsirE strains.

File name: Supplementary Data 5

Description: Numerical source data for graphs and charts
